# High-frequency harmonics suppression in high-speed railway through magnetic integrated *LLCL* filter

**DOI:** 10.1371/journal.pone.0304464

**Published:** 2024-06-03

**Authors:** Maged Al-Barashi, Yongjun Wang, Muhammad Shoaib Bhutta

**Affiliations:** 1 School of Aeronautics and Astronautics, Guilin University of Aerospace Technology, Guilin, China; 2 School of Automobile Engineering, Guilin University of Aerospace Technology, Guilin, China; Ghani Khan Choudhury Institute of Engineering and Technology, INDIA

## Abstract

The traction converter modulation generates switching-frequencies current harmonics. The trapped filters can eliminate these switching harmonics, reducing total inductance and filter size. Nonetheless, in comparison with the typical inductor-capacitor-inductor (*LCL*) filter, the trap inductor needs a larger magnetic core. Moreover, the trapped filter has not been analyzed in the traction systems. This paper proposes a magnetic integrated inductor-trap-inductor (*LLCL*) filter to decrease the filter’s size and investigate its application in traction converters. In fact, the application range of this filter is quite broad, and it can be used in various electrical power systems, including industrial power systems, renewable energy systems, transportation systems, and building power systems. The *LC*-trap may be formed by connecting the equivalent trap inductor, introduced through the magnetic coupling between inverter-side and grid-side inductors, in series with the filter capacitor. Furthermore, for H-bridge unipolar pulse width modulation (PWM) traction converters, the prominent switching harmonics are concentrated at the double switching frequencies. Therefore, the stability zone is expanded by moving the resonance above the Nyquist frequency. The presented filter’s features and design are thoroughly analyzed. The proposed method is finally validated by the MATLAB/Simulink simulation and hardware-in-the-loop (HIL) experimental results. Compared to the discrete windings, the integrated ones can save two magnetic cores. Furthermore, the proposed filter can meet IEEE criteria with 0.3% for all the harmonics and total harmonic distortion (THD) of 2.15% of the grid‐side current.

## 1. Introduction

The electric multiple units (EMUs) with pulse width modulation (PWM) traction converters are often used on China’s high-speed railway (HSR) lines [1]. For modern railway traction power-supply systems (TPSS), the undesired current harmonics produced by modulating traction inverters are a well-known issue [2]. Such harmonics may result in various problems that reduce the performance of the traction network, including torque bursts, severe mistakes and disturbances, and communications system interruptions [3]. On several electric railways, harmonic resonance occurrences have led to the faulty triggering of protection systems, breakdown of high-voltage installations, and maybe train blockages [4]. Thus, one of the critical issues for HSR lines is correcting for and resolving harmonic pollution. The harmonics at low frequencies may be suppressed using adequately constructed repeated controllers [5] or proportional-resonant ones [6]. However, the high-frequency harmonics, especially those at the switching frequency and its multiples, could be mitigated by passive filters [7]. When connected to the TPSS, traction converters must adhere to the grid codes.

The problem of harmonics at high frequencies in HSR is frequently addressed using the traction network or the high-speed train (HST) driving system. The traction harmonics reduction in power networks has received the majority of research attention. Several studies employ passive filters for changing the harmonic impedance at the traction network [7]. Capacitor filter (*C*) and inductor-capacitor filter (*LC*) are examples of these filters. This method is very costly in TPSS with high power and voltage. For the HST driving system, there is an alternative method to decrease the harmonics generated by the traction inverters. This approach attenuates the HSR high-frequency harmonics by using the selective harmonics elimination (SHE) PWM approach [4]. The offline optimal PWM approach is very sensitive to system parameters and requires complex computations to solve the fundamental equations. High-frequency harmonics may be easily absorbed with large filter inductances. Even yet, a straightforward approach like this will ultimately increase the system’s size, expenses, and controller bandwidths. Except for a little additional capacitor utilized in the manner given in [8], the well-known *LCL* filters thus replaced the conventional *L* ones, where the total value of inductance is the same.

Over the past several years, network-tied converters have increasingly used *LCL* filters to minimize harmonics at high frequencies in the input or output current of renewable energies [9]. On the other hand, boost inverter filtered by traditional *L* filter has been heavily utilized by the HSTs, particularly as a grid-side converter. To maximize iron core consumption and meet the requirements for compact size and lightweight in HST equipment, the leakage inductance of the traction transformer (TT) was often employed as an inductance of the grid-side converter [10]. However, the *LCL* filters have severe limitations, including poor switching harmonics absorption, significant passive components, and electrical network resonance [11]. Different *LCL*-modified filter configurations have been presented in [12–23], where most of them are with extra *LC* traps connected in parallel or series and resonated to the dominant switching frequency. These traps enable bypassing or blocking of particular harmonics. The inductance and capacitance of the filters, as well as the total harmonic distortion (THD), will all be decreased due to removing these harmonics from the current fed into the network. Compared to other novel passive filters, the inductor-trap-inductor (*LLCL*) filter introduced in [24–30] has lately drawn more interest. The series *LC* trap was employed to substitute the capacitor in the *LCL* filter, where it is resonated to the switching frequency.

Most prior research has been on lowering the total inductance of power filters to decrease their expense and size. The magnetic cores are principally responsible for the total size of the power filters. Trapped filters, however, include additional magnetic cores that significantly increase the system size despite a significant decrease in overall inductance. The magnetic integration strategy, which integrates several discrete inductors onto a single magnetic core, is the most popular solution for that issue [10,31–37]. To decrease the core size and magnetically integrate single- or three-phase *LCL* filters, an EIE-type core has been suggested in [32,38] to boost converter efficiency. In [39], it is proposed to integrate *LCL* filters with a composite delta-yoke core magnetically. This core is over 10% smaller than the EIE one. Magnetic integration always leads to magnetic coupling, notwithstanding the possibility of decoupled core topologies like EIE and delta-yoke types. The magnetic coupling impacts the harmonic absorption of the *LCL* filter because it adds coupling inductance to the filter capacitor branch. For minimizing the effects of magnetic coupling, [38,40] employed a good-permeable I-type core in the EIE one. However, this constrains the design’s flexibility and parameters’ modification.

Meanwhile, the induced resonance may cause system stability problems. Several damping approaches, including passive damping [14,23,41], and active damping [42–46], have been proposed to ensure system stability. The passive damping influences would be much higher in actual implementations when the equivalent series resistance (ESR) of passive elements commonly rises in the high frequencies. It is also possible to make the system stable using a single-current loop controller with an unavoidable delay by configuring the filter resonance frequency suitably [47–49]. However, designing the resonant frequency beyond the Nyquist frequency, i.e., half the sampling frequency *f*_*s*_/2, is chosen because of the additional losses of passive dampening techniques and the great sensors expenses of active dampening methods [11,50].

An innovative single-phase magnetic integrated *LLCL*-type PWM rectifier for HSTs having resonant frequency beyond the Nyquist frequency has been designed using the expertise gained from creating grid-connected rectifiers and filters for renewable energy sources. Furthermore, since the switching harmonics of H-bridge unipolar PWM converters emerge around the double switching frequency, this design is advantageous for these devices because it is more economical when the resonance is set at high frequencies. The inverter-side and grid-side inductances, along with a trap with zero impedance at the resonant frequency, make up an integrated *LLCL* filter. The trap in the proposed filter attenuates harmonics at the double switching frequency, which dominates current harmonics. Therefore, the required number of filter inductors may be reduced since the filter deals with more minor harmonics in the higher multiples of the switching frequency. To minimize the current harmonics at high frequencies, which may cause resonances in the TPSS, this filter is meant to take the place of the conventional *L* and *LCL* ones.

The area needed for *LCL*-type filter devices also has to be reduced because of the space restriction in HSTs for air-core inductors, which is solved by the proposed filter. The presented filter, according to simulation and hardware-in-the-loop (HIL) experimental results, may meet the power quality criteria and the constrained space in the locomotive. The two windings of the filter could be coiled on one magnetic core. A good design, applicability, and linearity of the inductor are advantages of the proposed technique.

To show the filter’s feasibility, In the subsequent sections, it is evaluated against some passive filters, i.e., its counterpart discrete *LLCL*, *LCL*, and *L* ones. Only three passive filters were utilized to reduce harmonics in traction systems, i.e., the traditional *L*, *LCL*, and integrated filtering inductors *LCL* ones [8,10,51]. Thus, the presented filter is just compared to the *LCL* and *L* ones. To confirm the magnetic integration’s effectiveness and the filter design’s adaptability, the presented filter is also compared to the discrete *LLCL* one. Compared to the previous techniques, the model, analysis, and verification results reported in this paper demonstrate that the adopted one can minimize the needed passive components for the magnetic integrated *LLCL* filter, resulting in a more cost-effective design. Furthermore, the conventional *LCL* filter performs poorly at low-frequency harmonics. Moreover, the discrete *LLCL* filter requires an extensive size and is also sensitive to resonance. Therefore, the selection of the proposed filter has been guided by its advantages compared with the other filters. Thus, the integrated *LLCL* filter can be considered suitable for operating in traction network systems since this filter has a robust structure. The advantages of the presented filter can be summarized by:

1. reducing the size by winding many coils through magnetic integration,
2. attenuating the switching frequency and its multiple harmonics that reduce the current THD,
3. assuring durability against the filter parameters differences, and
4. preserving system stability under transient conditions.

Furthermore, SHE [52,53], which may eliminate selected harmonics using the controller system, has been compared with the proposed approach. SHE is only adequate for offline calculations, which calls for more detailed lookup tables at the low fundamental frequency and attenuates harmonics at low frequencies to increase ensuing harmonics at high frequencies [54–57]. In contrast, the proposed method is straightforward and able to achieve the SHE’s influence at low harmonics. In addition, the output waveforms are enhanced, the undesired harmonic distortion is removed, and the converter losses are decreased. Without using any extra elements, the *LCL* filter’s capacitor with the equivalent inductance created by the coupling effect between the two inductances produces the *LC* trap. The two inductors are also held together by a single magnetic core, which minimizes the total size and related expenses.

The research work has innovative ideas and a certain degree of theoretical innovation. The main innovations of this paper are as follows:

1. To decrease the large size of the conventional filters, this paper proposes designing a new passive filter, called the magnetic integrated *LLCL* filter, to attenuate the current switching harmonics with minimized size. In the proposed filter, two discrete windings are integrated on one magnetic core. The proposed filter is effective in reducing the harmonics to the standard allowed limits with a reduction of the filter size compared with the conventional passive filters.
2. To assess the resonance issue in passive integrated filters, this paper proposes establishing the magnetic integrated *LLCL* with resonance frequency beyond Nyquist frequency since. The proposed filter can suppress the current harmonics distortions and resonance with a small size.
3. To improve the problems in conventional solutions of harmonics mitigation in the traction converters, represented by the small area for large air-core inductors in the HST and weak harmonics suppression, this paper proposes an optimized integrated filter in the traction converters. The proposed integrated passive filter is applicable in the HSR traction converters for harmonics suppression and performance under different operation conditions.

The contributions of the paper are listed below:

1. An integrated *LLCL* filter with resonance beyond the Nyquist frequency is designed, simulated, and validated step-by-step.
2. A detailed analysis of the magnetic integration idea of many inductors in traction converters has been done.
3. The harmonics removal and size decrement are utilized to validate the proposed method.

**Fig 1:**
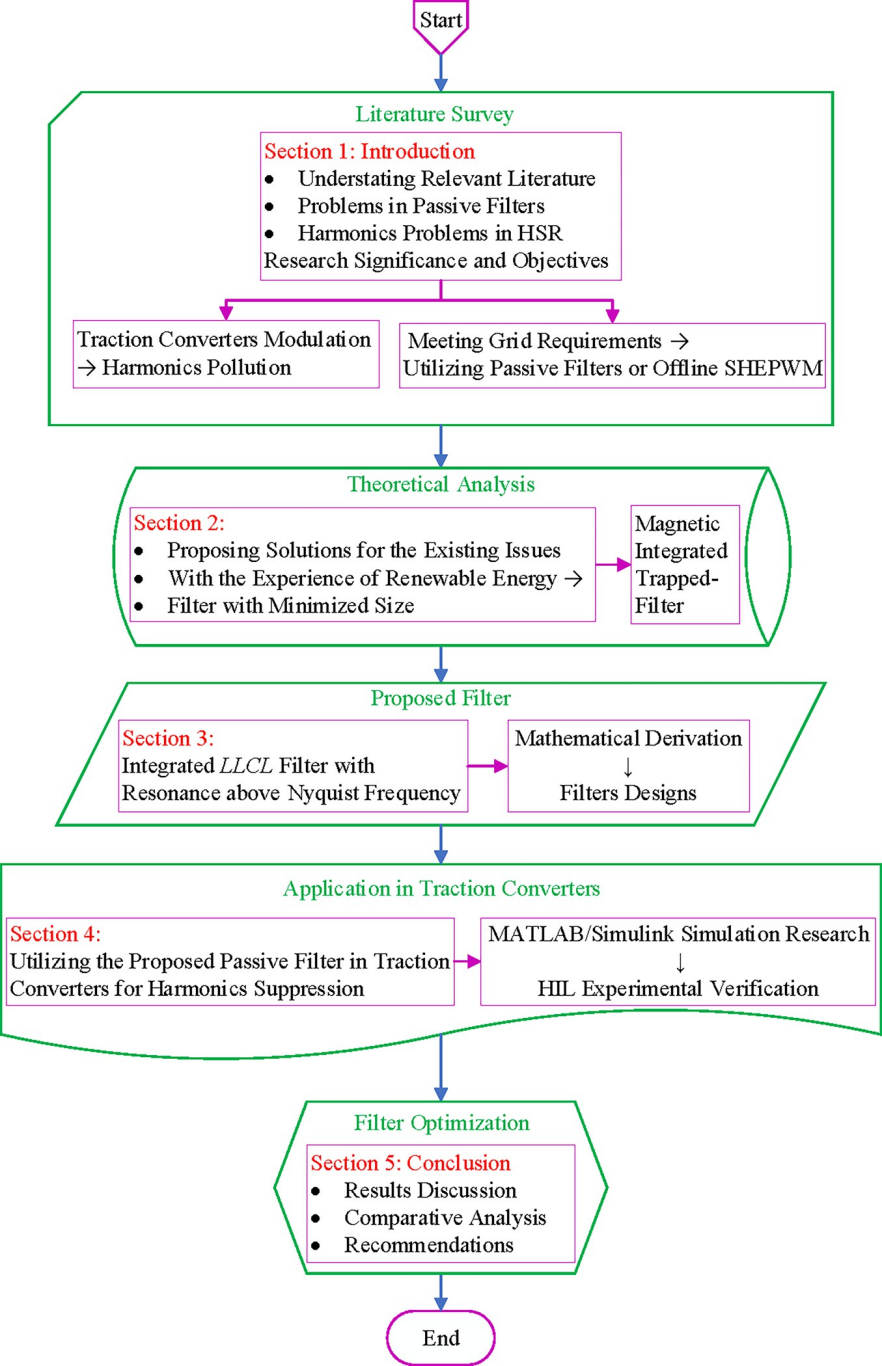
Paper outline processes and technical route.

The remainder of the article is arranged in the following manner. Section 2 will initially display the system structure of the integrated *LLCL* filter before studying the magnetic circuit. Section 3 presents the integrated *LLCL* parameters designing and modeling. Results from simulations and HIL experiments are presented in Section 4 to evaluate the feasibility of the filter. Lastly, Section 5 brings the article to the conclusion. This paper structure is shown in the flow chart in Fig 1, showing the processes and technical route.

## 2. System structure and analysis of magnetic circuit

### 2.1. System structure

**Fig 2:**
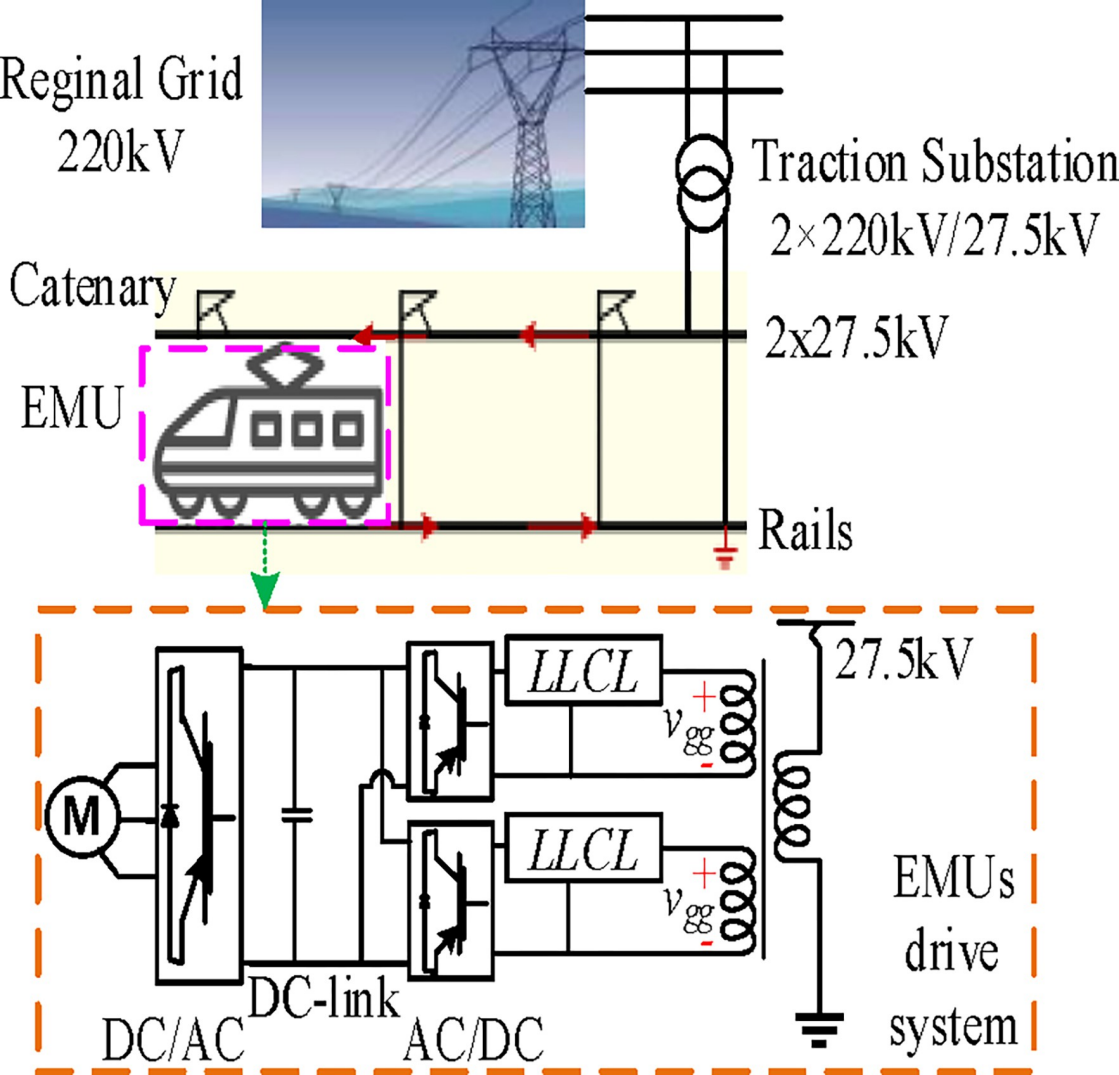
Structure of traction power supply substation.

The standard TPSS structure utilized in China’s HSR is shown in Fig 2 [8]. To provide the all-parallel autotransformer (AT)-fed network via a V/x-structure TT, the three-phase 220 kV network voltage must be reduced to 27.5 kV single-phase double-feeders in the electrical supply substation. TTs may be connected using single-phase, Scott, V/v(V/x), and Ynd11, among other methods. The V/x-structure TT is used by German and Chinese HSR due to its excellent capacity utilization, straightforward wiring, and compatibility with AT catenary systems [1]. The all-parallel AT-fed TPSS grid architecture is commonly used in heavy-duty trains and HSR around the world due to its advantages of extended power supply, minimal voltage drop, and electromagnetic interference [31]. The ATs, mounted at the AT substation or section post, are spaced along the track by around 10 to 15 kilometers. In the complex AT traction electrical network, the feeders, contact lines, rail, protection wires, communication cables, and integrated grounding cables are considered multiconductor transmission lines.

Fig 2 shows a single-phase inverter drive system for an equivalent HST. The line-side inverter in every HST’s power grid comprises two PWM rectifiers connected in parallel. Two interlaced two-level single-phase converters are used in the power conversion stage to achieve a high power factor and current carrying capacity. As presented in [1], the train inverters are frequently two-level, multi-interlaced, two- or three-level. Two interlaced two-level PWM inverter driving system of EMUs and all-parallel AT-fed network structures are used for analysis in this paper. However, there are several other grid modes and EMUs kinds, as was already indicated. However, the findings of the simulation and HIL experiments and the established rule of the researched filter are applicable for the other types, opening up the possibility of multiple distinct lines of future study.

As this paper mainly focuses on the application of the magnetic integrated *LLCL* filter, the detailed mathematical model of HSR and initial parameters are not presented here, which has already been elaborated in existing studies [1,2,7,8,58–60]. For example, a uniform mathematical model of TPSS and China HST is presented in [58] using the nodal admittance matrix. The HSR modeling can potentially affect the filtering effect of the passive filter. The modeling of the HSR system, including the power converters, power cables, and load, can affect the performance of the passive filter in several ways. For example, the impedance of the load and the power cables can influence the filter’s resonant frequency, which can, in turn, affect its ability to attenuate harmonics.

Additionally, the type and level of nonlinearities presented in the HSR system can affect the harmonic content of the signals transmitted through the filter, which can also impact its performance. As described in Fig 2, the passive filter is placed in the PWM-controlled train to suppress the harmonic generated by the traction converter since it is the origin of harmonics in the low voltage side of the TT. Therefore, the traction grid is seen from the filter as an ideal linear component, with *v*_*gg*_ representing the contact-line voltage at the train location, while the equivalent load is seen as a fixed resistance [2,8,10,60]. Thus, to simplify the analysis, the values of the EMUs’ converter parameters affecting the filter design and performance are listed in Section 3.

**Fig 3:**
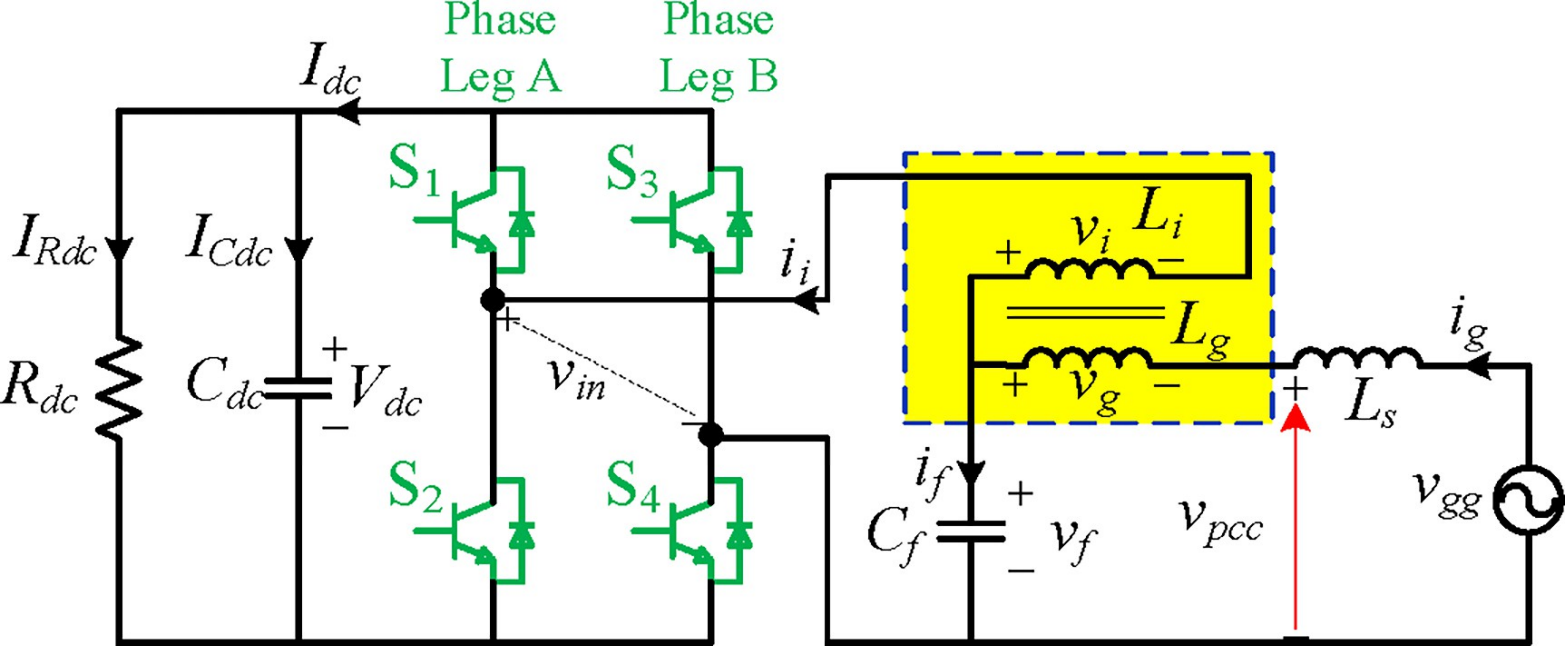
H-bridge traction converter with int. *LLCL* filter.

Fig 3 shows the harmonics assessment circuit from the traction electrical network to the line-side traction converter with an integrated *LLCL* filter. Here, just a single integrated *LLCL* inverter is used for simplification. With a 27.5/1.55 transformation ratio, the TT is regarded as a standard linear component. The equivalent inductor of the secondary side of the TT is represented by *L*_*s*_. It should be noted that this specific arrangement does not significantly limit the use of trapped filters. These filters may often be used in dc/ac or ac/dc generation systems that are either single-phase or three-phase, standalone or grid-connected [35].

In this case, *R*_*dc*_ is the equivalent load of a single integrated *LLCL* inverter in the traction inverter-motor driving system, *C*_*dc*_ is the dc-link capacitor, and *I*_*Rdc*_ and *I*_*Cdc*_ denote their currents. Four switches designated as *S*_1_, *S*_2_, *S*_3_, and *S*_4_ that operate at the switching frequency *f*_*sw*_ convert the dc-link voltage *V*_*dc*_ into ac voltage *v*_*in*_ that contains harmonics at the prominent switching frequency 2*f*_*sw*_ and its multiples [61]. The harmonics are concentrated around 2*f*_*sw*_ when using a unipolar sinusoidal PWM (SPWM). Thus, 2*f*_*sw*_ is the actual switching frequency. It is also important to remember that harmonics at the double switching frequency 2*f*_*sw*_ are much more prominent than those at high switching frequencies, which have the most output current harmonics. For this reason, the total harmonics can be effectively decreased by eliminating the harmonics at 2*f*_sw_. In real situations, one *LC*-trap is favoured due to its lower expense and size, and this is the approach that is further investigated in this work.

As discussed above, one of the biggest challenges for HSR operators is to keep the power quality at a high level by limiting the line voltage and currents’ THD at the point of common coupling (PCC). Since the current in these non-linear systems is still periodic (just not sinusoidal), this change in the nature of the current can be described in terms of the harmonic distortion of the current. Because the grid is assumed to provide a sinusoidal voltage, then there is no voltage at frequencies other than the fundamental. Therefore, only current harmonics must be continuously monitored for power quality. THD is a common measurement of the level of harmonic distortion present in electrical power networks. The THD term is expressed as the effective value of all harmonics divided by the effective value of its fundamental current. The distortion as a percentage of THD is defined as in (1), where *I*_*n*_ is the effective current of the n^th^ harmonics and *I*_*1*_ is the effective current of the fundamental frequency. If the harmonic components are equal to the “0”, then THD will be equal to the “0”. The presence of harmonic currents and voltages in the power system means the distortion of sinusoidal waves. Deteriorated waves are called as non-sinusoidal waves. Voltage and current waveform distortion due to harmonics can lead to the high-voltage installations and HST either damaged or blockaded [62–65].


$$THD=\frac{\sqrt{\sum_{n=2}^{\infty }I_{n}^{2}}}{I_{1}}.$$ (1)

The TT might be viewed as a typical transformer. To create the worst instability situation, the equivalent series resistors of the filter components are disregarded. The inverter-side inductor *L*_*i*_ and grid-side inductors *L*_*g*_ are connected in series, where their voltages are *v*_*i*_ and *v*_*g*_, respectively, and their currents are *i*_*i*_ and *i*_*g*_. At the same time, the dc bus current is represented by *I*_*dc*_. A shunt filter capacitor *C*_*f*_ has been inserted at the connection point of *L*_*i*_ and *L*_*g*_. The voltage at this point is denoted by *v*_*f*_, whereas *i*_*f*_ represents the current flowing through *C*_*f*_. In addition, *v*_*pcc*_ represents the voltage at the PCC. At this point, the inverter is tied to the grid.

The traction grid conditions may considerably impact the passive filter filtration effect. The traction network is assumed to be weak throughout this paper’s discussion, which means a low short-circuit ratio. As a result, the system impedance could change significantly [66]. For the worst stability case, an idealized voltage source with series inductance, denoted by *L*_*s*_ in Fig 3, is used to represent the grid. Since the network resistance would reduce the resonant peaks, the focus will only be on the impact of the network inductance.

Moreover, when several converters operate simultaneously to share power, the equivalent grid inductance detected by one converter is proportional to the number of inverters. The network impedance significantly impacts as the number of inverters rises [34]. According to [11], higher *L*_*s*_ may weaken the resonant poles. The filter was shown to undergo brief transient oscillations before returning to its setpoint. Despite this, compared to the strong grid circumstances, the system’s dynamic reaction took longer.

### 2.2. Proposed magnetic integration approach of *LLCL* filter

The proposed magnetic integration method for an *LLCL* filter will be covered in this section. In-depth discussions of the passive filter inductors’ design ideas may be found in the pertinent literature. The grid-side and inverter-side inductors in a typical *LCL* filter require the fabrication of two inductors since each has its own specific inductor. Assume that the *LLCL* filter [24–30], i.e., the equivalent discrete filter to the integrated *LLCL* one, as illustrated in Section 2.3, uses one *LC*-trap. In such an instance, in addition to the grid-side and inverter-side inductors, one more inductor must be fabricated. As a result, the discrete *LLCL* filter still has a big size and high expenses due to the requirement of another magnetic core for the trap inductor. This is still the case even if the total inductance is less than that of a conventional *LCL* filter. It is advised to apply magnetic integration, an excellent method that has already been employed in grid-tied converters for *LCL* [38,40], and *LLCL* [11,67] filters, to further increase the power density while minimizing expenses.

**Fig 4:**
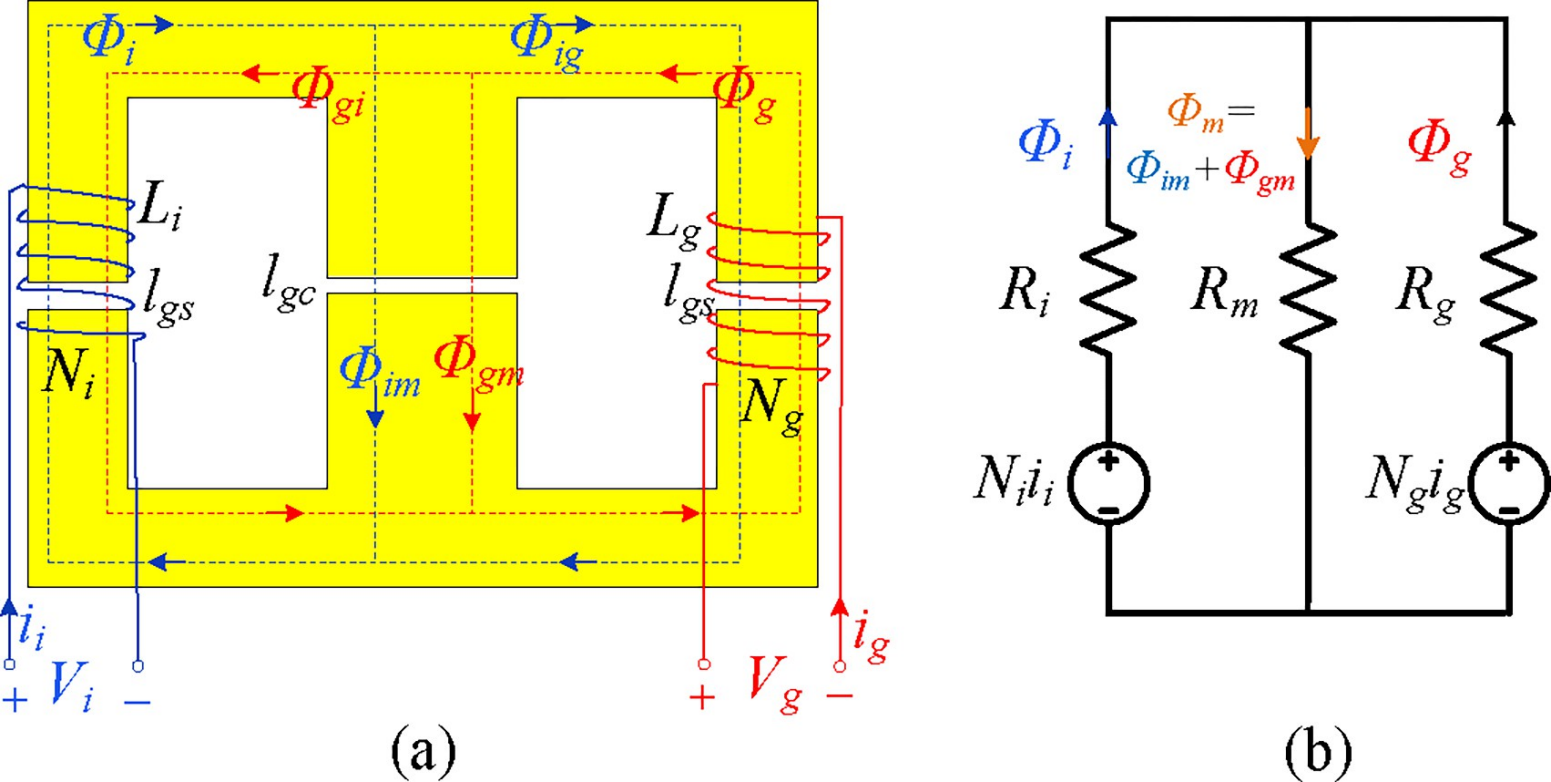
Proposed magnetic integration of *LLCL* filter.

**Fig 5:**
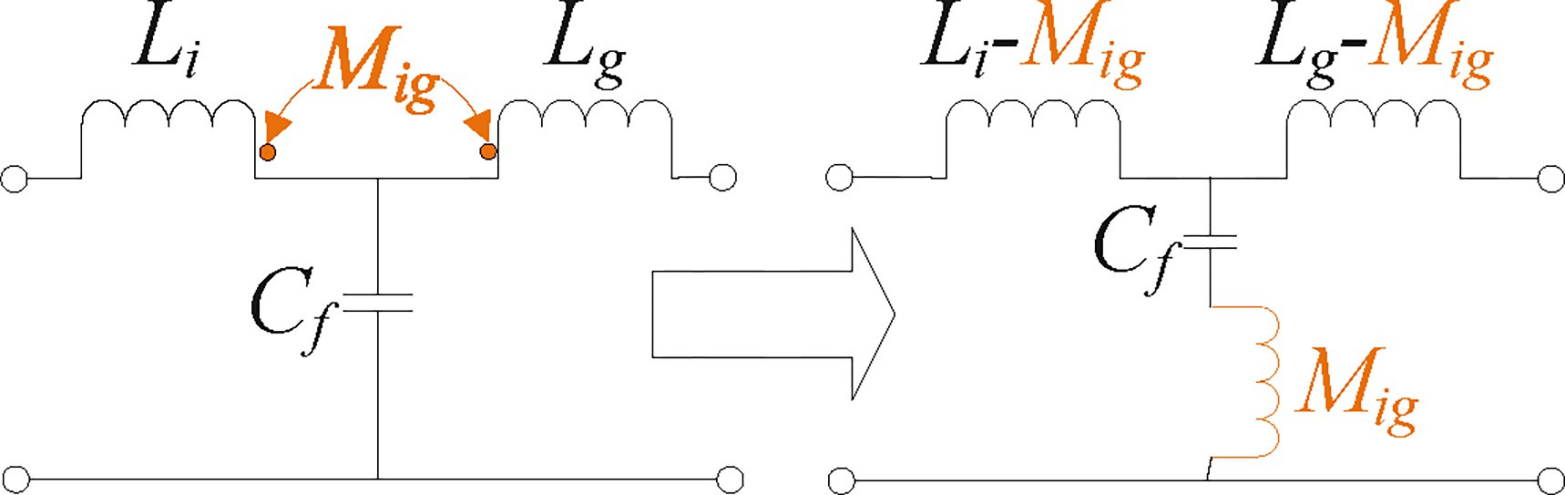
Circuit diagram of an integrated *LLCL* filter.

Fig 4(A) illustrates the magnetic integration for the *LLCL* filter. In contrast, Fig 5 depicts the electric connection schematic for such a circuit. By winding *L*_*i*_ and *L*_*g*_, with winding turns *N*_*i*_ and *N*_*g*_, on the side limbs, the coupling influence may be fully used to lower the filter size. In such a magnetic core, the *L*_*i*_ and *L*_*g*_ windings are negatively coupled because the flux directions at the side limbs are opposite. The central concept of the presented magnetic integration is constructing a trap-inductor via the magnetic coupling between *L*_*i*_ and *L*_*g*_ inductors called an active trap inductor. In the proposed design, integrating *L*_*i*_ and *L*_*g*_ introduces a coupling inductor for the *LC* trap. Based on Fig 4(B), *Φ*_*i*_ refers to the flux generated by *L*_*i*_ winding. In contrast, *Φ*_*im*_ and *Φ*_*ig*_ are the fluxes generated by *L*_*i*_ winding and flowing across the central limb and *L*_*g*_ winding, respectively, as described in (2), where *R*_*i*_, *R*_*m*_, and *R*_*g*_ can refer to the magnetic resistors of the three limbs, respectively [11,34,35,68].


$$\left\{ \begin{array}{l} \Phi_{i}=\frac{N_{i}i_{i}(R_{g}+R_{m})}{R_{i}R_{m}+R_{i}R_{g}+R_{m}R_{g}},\\ \Phi_{im}=\frac{N_{i}i_{i}R_{g}}{R_{i}R_{m}+R_{i}R_{g}+R_{m}R_{g}},\\ \Phi_{ig}=\frac{N_{i}i_{i}R_{m}}{R_{i}R_{m}+R_{i}R_{g}+R_{m}R_{g}}. \end{array}\right.$$ (2)

Furthermore, the flux generated by *L*_*g*_ winding is *Φ*_*g*_, where the fluxes generated by *L*_*g*_ and flowing across the central limb and *L*_*i*_ winding are *Φ*_*gm*_ and *Φ*_*gi*_, respectively, and it is easy to explain them in (3) [11,34,35,68].


$$\left\{ \begin{array}{l} \Phi_{g}=\frac{N_{g}i_{g}(R_{i}+R_{m})}{R_{i}R_{m}+R_{i}R_{g}+R_{m}R_{g}},\\ \Phi_{gm}=\frac{N_{g}i_{g}R_{i}}{R_{i}R_{m}+R_{i}R_{g}+R_{m}R_{g}},\\ \Phi_{gi}=\frac{N_{g}i_{g}R_{m}}{R_{i}R_{m}+R_{i}R_{g}+R_{m}R_{g}}. \end{array}\right.$$ (3)

Each inductor winding’s total flux consists of the self-flux and the mutual flux.‎ *V*_*i*_ and *V*_*g*_ are determined as in (4) [11,68]. They could either be determined, according to (2)–(4), as in (5) [11,35]. The self-inductances *L*_*i*_ and *L*_*g*_ and the mutual inductances *M*_*ig*_ and *M*_*gi*_ could be described as in (6) [11,34,35,67].


$$\left\{ \begin{array}{l} V_{i}=N_{i}\frac{d}{dt}(\Phi_{i}-\Phi_{gi}),\\ V_{g}=N_{g}\frac{d}{dt}(\Phi_{g}-\Phi_{ig}). \end{array}\right.$$ (4)

$$(\begin{array}{l} V_{i}\\ V_{g} \end{array})=(\begin{array}{cc} L_{i} & -M_{ig}\\ -M_{gi} & L_{g} \end{array})(\begin{array}{l} \frac{di_{i}}{dt}\\ \frac{di_{g}}{dt} \end{array}),$$ (5)

$$\left\{ \begin{array}{l} L_{i}=\frac{N_{i}^{2}(R_{g}+R_{m})}{R_{i}R_{m}+R_{i}R_{g}+R_{m}R_{g}},\\ L_{g}=\frac{N_{g}^{2}(R_{i}+R_{m})}{R_{i}R_{m}+R_{i}R_{g}+R_{m}R_{g}},\\ M_{ig}=M_{gi}=\frac{N_{i}N_{g}R_{m}}{R_{i}R_{m}+R_{i}R_{g}+R_{m}R_{g}}. \end{array}\right.$$ (6)

It must be indicated that the *M*_*ig*_ and *M*_*gi*_ are equal and thus can be referred to as *M*_*ig*_. The two inductors appear to be coupled according to (5) and (6), so (7) could represent *V*_*i*_ and *V*_*g*_ [11,67].


$$\left\{ \begin{array}{l} V_{i}=V_{in}-V_{f}=L_{i}\frac{di_{i}}{dt}-M_{ig}\frac{di_{g}}{dt},\\ V_{g}=V_{f}-V_{pcc}=L_{g}\frac{di_{g}}{dt}-M_{ig}\frac{di_{i}}{dt}. \end{array}\right.$$ (7)

The self and mutual inductances are, based on (6), determined by the magnetic resistances that could be defined by (8). In this equation, the cross-sectional areas of the central and side limbs are represented by *A*_*C*_ and *A*_*S*_, with *A*_S_ = 1/2*A*_C_. *l*_*gc*_ and *l*_*gs*_ represent the air gap lengths of the central and side limbs. Moreover, *μ*_0_ = 4*π* × 10^−7^ N / A^2^ refers to the air permeability [11,34,35,67,68]. In the end, (9) is used to calculate the self and mutual inductances by substituting (8) into (6) [11,34,35]. As illustrated in (10), the ratio of the mutual inductance to the square root of the product of the self-inductances is known as the coupling coefficient *k*_*Mig*_. Furthermore, it can be found that by adjusting the air gap lengths *l*_*gs*_ and *l*_*gc*_, i.e., the ratio *l*_*gs*_/*l*_*gc*_, the coupling coefficient could be tuned effectively.


$$\left\{ \begin{array}{l} R_{i}=R_{g}=\frac{l_{gs}}{A_{S}\mu_{0}},\\ R_{m}=\frac{l_{gc}}{A_{C}\mu_{0}}. \end{array}\right.$$ (8)

$$\left\{ \begin{array}{l} L_{i}=\frac{N_{i}^{2}\mu_{0}(l_{gc}A_{S}+l_{gs}A_{C})}{2l_{gs}(l_{gc}+l_{gs})},\\ L_{g}=\frac{N_{g}^{2}\mu_{0}(l_{gc}A_{S}+l_{gs}A_{C})}{2l_{gs}(l_{gc}+l_{gs})},\\ M_{ig}=\frac{N_{i}N_{g}\mu_{0}l_{gc}A_{S}}{2l_{gs}(l_{gc}+l_{gs})}. \end{array}\right.$$ (9)

$$k_{Mig}=\frac{M_{ig}}{\sqrt{L_{i}L_{g}}}=\frac{1}{1+2l_{gs}/l_{gc}}.$$ (10)

The magnetic coupling between two windings may be used in the approach method to create an equivalent trap inductor, as shown in Section 2.3. As a result, it is feasible to eliminate the requirement for two magnetic cores and reduce the expense and size of the *LLCL* filter. Furthermore, it is noted that there is no additional component in contrast to the discrete *LLCL* filter.

### 2.3. Properties and filtering effectiveness of the proposed filter

The integrated *LLCL* filter’s circuit construction is shown in Fig 5, with an inverter-side inductance *L*_*i*_, a grid-side inductance *L*_*g*_, and an *LC* trap (*M*_*ig*_-*C*_*f*_). This *LC* trap resonates at 2*f*_*sw*_, which is the dominant switching frequency. To reduce the switching harmonics, the mutual inductor *M*_*ig*_ is used in the integrated *LLCL* filter to produce the resonant tank with *C*_*f*_. The resonant tank removes the output current’s particular harmonics. The whole filter attenuates the other harmonics at the same time. The integrated *LLCL* filter is designed using a method described in this work that involves setting the series resonant frequency to 2*f*_*sw*_.

**Fig 6:**
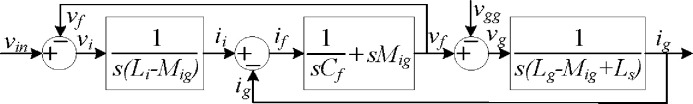
Integrated *LLCL* filter block diagram.

Furthermore, Fig 6 presents the block diagram of the proposed filter. From *v*_in_ to *i*_*g*_, the integrated *LLCL* filter’s transfer function *G*_*LLCL*_(*s*) may be derived as in (11), where the coefficients are presented in the S1 Appendix.


$$G_{LLCL}(s)=\frac{i_{g}(s)}{v_{in}(s)}=\frac{a_{2}s^{2}+1}{b_{3}s^{3}+b_{1}s}$$ (11)

It can be found that *G*_*LLCL*_(*s*) has a zero at *ω*_*t*_ = (1/(*C*_*f*_*M*_*ig*_))^1/2^, where *ω*_*t*_ represents the trap angular frequency. Setting this frequency to the dominant switching frequency, as shown in (12), where *f*_*t*_ is the trap frequency, will effectively suppress the corresponding switching harmonics. Furthermore, simulation and HIL experimental results will be used in Section 4 to confirm the THD reduction and validity of the proposed filter.


$$f_{t}=2f_{sw}=\frac{1}{2\pi }\sqrt{\frac{1}{a_{2}}}=\frac{1}{2\pi }\sqrt{\frac{1}{C_{f}M_{ig}}}$$ (12)

**Fig 7:**
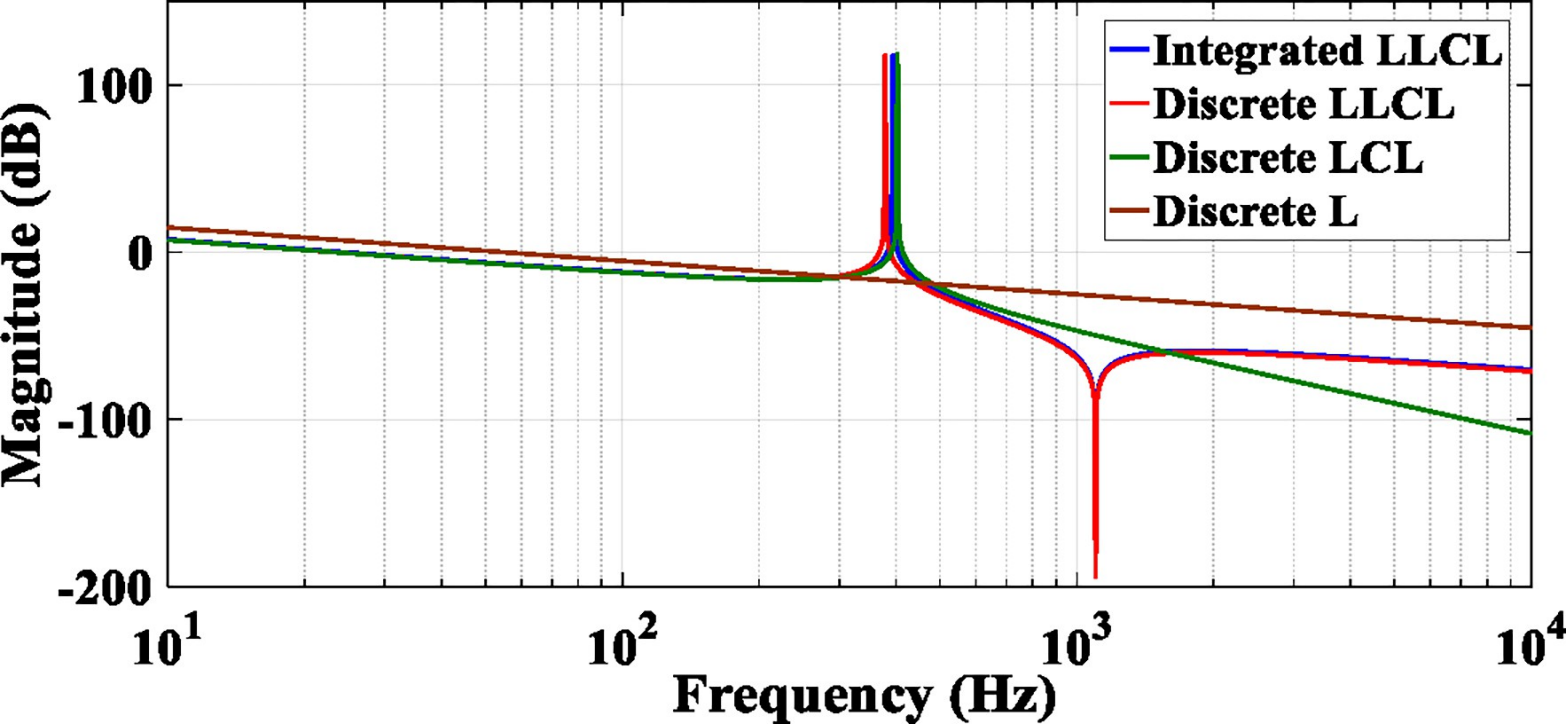
Bode diagrams for the four filters.

Employing the values in Table 1, Fig 7 displays the Bode diagrams *i*_*g*_(*s*)/*v*_*in*_(*s*) of the integrated *LLCL* and discrete *LLCL*, *LCL*, and *L* filters. These values are derived based on [8]. A detailed explanation of the procedures utilized to design these parameters is provided in Section 3. The integrated *LLCL* filter maintains the discrete *LLCL* filter’s characteristics, as shown in Fig 7, and produces a potent harmonics suppression at 2*f*_*sw*_, where this frequency has a magnitude trap. Furthermore, Fig 7 shows that the magnitude-frequency characteristics of *G*_*LLCL*_(*s*) have a conjugate resonant peak because the degree of their denominator is 3. This resonant peak is obtained by putting the denominator of *G*_*LLCL*_(*s*) in (11) equals zero, substituting *s* with *jω*. The integrated *LLCL* filter’s resonance frequency *f*_*r*_ can be determined using (13) [11,67].


$$f_{r}=\frac{1}{2\pi }\sqrt{\frac{b_{1}}{b_{3}}}=\frac{1}{2\pi }\sqrt{\frac{L_{i}+L_{g}-2M_{ig}+L_{s}}{C_{f}(L_{i}-M_{ig})(L_{g}-M_{ig}+L_{s})}}.$$ (13)

**Table 1:** Parameters values of the filters.

Filter type	Integrated *LLCL*	Discrete *LLCL*	Discrete *LCL [8]*	Discrete *L*
*L*_*i*_(*mH*)	1.63	1.63	1.63	2.93
*L*_*g*_(*mH*)	1.3	1.3	1.3	-
*C*_*f*_(*μF*)	125	125	125	-
*L*_*f*_(*mH*)	-	0.167	-	-
*M*_*ig*_(*mH*)	0.167	-	-	-
*k*_*Mig*_	0.115	-	-	-

The discrete *LCL* filter has the highest roll-off rate of -60 dB/dec at the high-frequency domains, as shown in Fig 7, but since it lacks a trap magnitude, its suppression at the switching frequency is less. After the trap frequency, the proposed integrated *LLCL* filter has a harmonics suppression of -20 dB/dec. The resonant peaks may cause system stability problems. Designing the resonant frequency beyond the Nyquist frequency is adopted in this paper for assuring the system stability and exploring the new stability zone, which can be derived as in (14).


$$\frac{f_{sw}}{2}<f_{r}<\frac{5f_{sw}}{6}.$$ (14)

## 3. Integrated *LLCL* filter design and modeling

The parameters design of the proposed filter for traction converters is covered in this section. The *L*_*s*_ has been adjusted at 4 mH. The integrated *LLCL* filter parameters could be designed using the system parameters listed in Table 2 considering the criteria of the harmonic restrictions permitted by IEEE 519–2014 [8,69,70], the inverter-side current ripple Δ*I*_*Li*_ less than 40%, and utilizing below 5% of the reactive power. According to *R*_*dc*_, the inverter works as a rectifier here. Moreover, designing *L*_*i*_, which may be done using the *LCL* filter’s designing approach [8], is the first step in the design process of the integrated *LLCL* filter. Consequently, with Δ*I*_*Li*_ ≤ 40%, *L*_*i*_ is designed as in (15) for an H-bridge single-phase unipolar SPWM rectifier.


$$L_{i}=\frac{0.5\times 0.5\times V_{dc}}{2f_{sw}\Delta I_{Li}}\approx 1.63mH.$$ (15)

**Table 2:** Parameter values for the EMUs inverter.

Description	Symbol	Value
Contact-line voltage (RMS)	*V*_*gg*_	1550 V
System rated power	*P*_*o*_	900 kW
DC-link capacitor	*C*_*dc*_	4 mF
Load resistor	*R*_*dc*_	10 Ω
DC-link voltage	*V*_*dc*_	3000 V
Switching frequency	*f*_*sw*_	550 Hz
Fundamental frequency	*f*_*o*_	50 Hz
Network inductor	*L*_*s*_	4 mH

Design *L*_*g*_ is similar to *L*_*i*_ to obtain the maximum amount of inductor utilization since such a system may attain the lowest resonance frequencies [36,37,50,71]. However, *L*_*g*_ has been reduced to 1.3 mH because the maximum and ripple currents have dropped by almost 40%.

It is necessary to check the total inductance *L*_*total*_ = *L*_*i*_ + *L*_*g*_ in order to keep the inductors’ ac voltage drop lower than the 10% root-mean-square (RMS) value of *v*_*gg*_ [72].

When selecting the filter capacitor *C*_*f*_, It is necessary to strike a balance between reactive power consumed at the fundamental frequency and the elimination of harmonics at high frequencies [8,73]. *C*_*f*_ can be as low as 125 F while considering the permitted reactive power. However, the total capacitance *C*_*total*_ = *C*_*f*_ must be limited by the amount of reactive power used under the rated conditions to satisfy the power factor criteria. Moreover, the reactive current will be excessive if *C*_*total*_ is higher than 0.05 p.u [8,50]. The increase in filter inductance might solve this problem.

Moreover, *M*_*ig*_ is designed by meeting (12)–(14), which means that the filter’s *f*_*r*_ must fall between 1/2*f*_*sw*_ and 5/6*f*_*sw*_, while *f*_*t*_ must equal 2*f*_*sw*_ = 1.1 kHz. *f*_*r*_ = 2/3*f*_*sw*_ = 367 Hz is chosen as the median value to increase stability and mitigate the loss of inductance with increasing current [11,50]. As a result, *M*_*ig*_ has been determined to be 0.167 mH.

**Fig 8:**
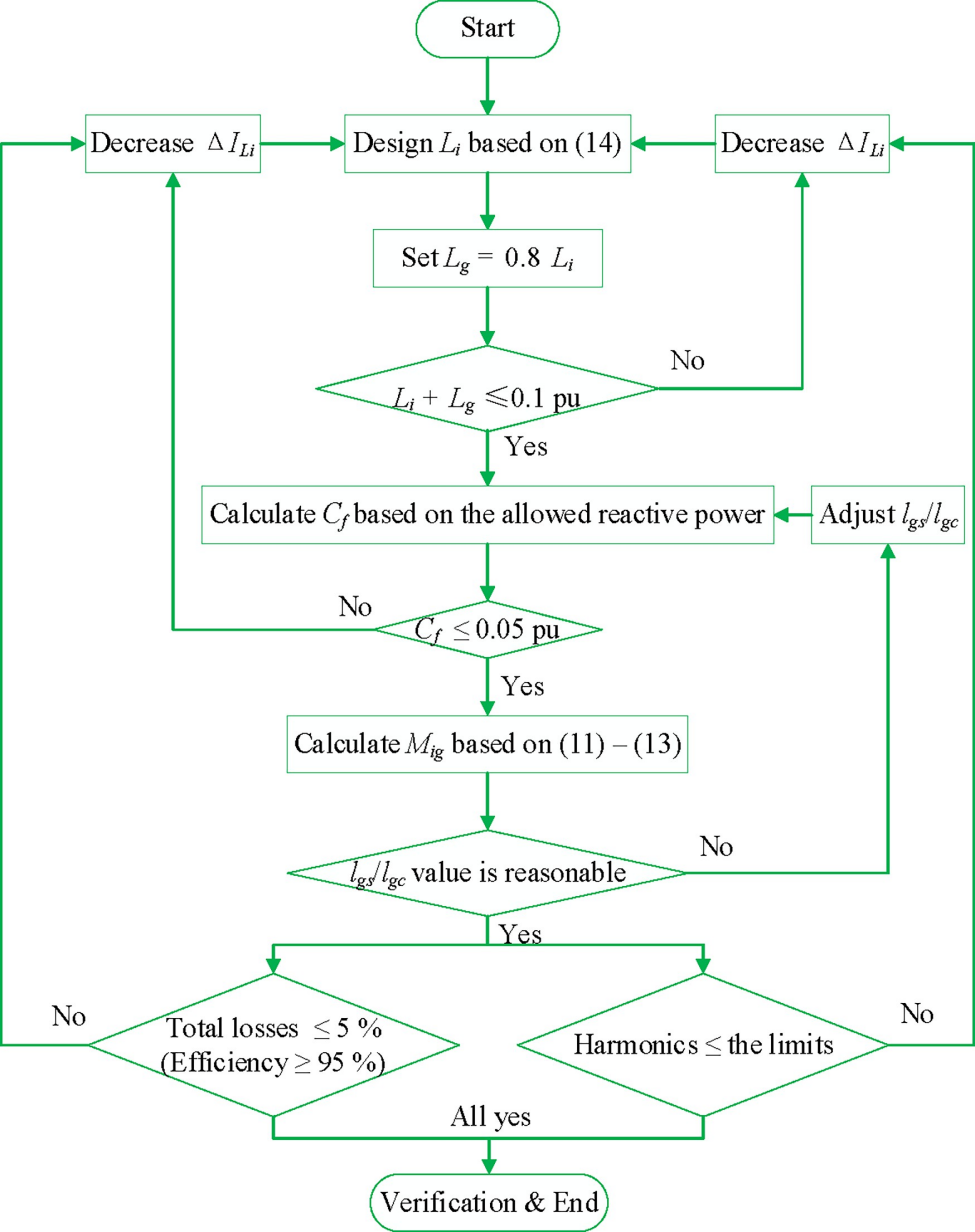
Flowchart for the int. *LLCL* filter design method.

Furthermore, *k*_*Mig*_, the pertinent coupling coefficient, is optimized to be 0.115 based on (10). Thus, the ratio of air gaps could be calculated as *l*_*gs*_/*l*_*gc*_ = 3.85, demonstrating that *L*_*i*_ and *L*_*g*_ are determined by *l*_*gs*_. In addition, Fig 8 displays the presented filter’s comprehensive designing flowchart.

The discrete *LLCL* filter is designed using a similar procedure, which implies that *L*_*i*_, *L*_*g*_, and *C*_*f*_ equal those of the proposed filter for providing a reasonable evaluation. The additional inductor *L*_*f*_ is determined by (12), replacing *M*_*ig*_ with *L*_*f*_, which is designed to be 0.167 mH.

By adopting an identical procedure, the *LCL* filter is designed, implying that *L*_*i*_, *L*_*g*_, and *C*_*f*_ are similar to the presented filter to give a consistent evaluation.

Additionally, the *L* filter may be identical to the *L*_*total*_ of the proposed filter or equal to the *LCL* filter with setting *C*_*f*_ = 0 *μ*F.

The parameters of the integrated *LLCL*, discrete *LLCL*, *LCL*, and *L* filters may be found in Table 1 following the application of these design processes. Furthermore, the traction converter with the proposed filter may be controlled similarly to that described in [35,51,54,60,74]. Thus, it will not be discussed here. For demonstrating the validity of the presented filter’s design, Section 4 will give the verification results.

As noticed, the proposed filter may create an equivalent extra inductor, saving one inductor and all of its parts. Additionally, one magnetic core may be saved by the proposed magnetic integration. This is because, unlike the discrete *LLCL* filter and other versions like *LTCL* and *LCL*-*LC* filters, which employ three magnetic cores, *L*_*i*_ and *L*_*g*_ might be coiled on the side limbs of a single magnetic core.

It is essential to compare the sizes of integrated inductors with discrete inductors. The discrete inductors of the *LLCL* filter could be, respectively, wound on the central limbs of three cores. To enable proper comparison, the filter inductances of the integrated and discrete *LLCL* filters are configured similarly. The biggest filter is the discrete *LLCL* filter, which has three cores. While the integrated *LLCL* and discrete *L* filters each have one core, and the discrete *LCL* filter has two cores.

## 4. Simulation and HIL experimental verification

With the parameters listed in Tables 1 and 2, the configuration in Fig 3 was used to develop MATLAB/Simulink simulation and HIL models. The objective is to evaluate the efficacy of the presented filter design technique and to contrast it with the separate *LLCL*, *LCL*, and *L* filters. It must be kept in mind that an adaptive proportional-resonant controller was employed in the traction converter control system that is not discussed in this article. This research employs the symmetrical, unipolar, regular sampled SPWM.

The advantages and disadvantages of several passive filters are compared, along with their robustness, complexity, and size. To investigate the efficiency, transient performance, and harmonics attenuation ability of four filters, simulations and HIL tests are performed. The various performance indexes that have been computed are listed in Table 3.

**Table 3:** Performance indicators of the filters.

Index	Integrated *LLCL*	Discrete *LLCL*	Discrete *LCL*	Discrete *L*
Dc Voltage fluctuation (V)	±140	±145	±150	±130
Size (number of cores)	1	3	2	1
Power losses (kW)	23.34	22.72	24.31	N/A
Harmonics around 2*f*_*sw*_ (% *I*_*ref*_)	0.02	0.01	0.10	0.19
Harmonics around 4*f*_*sw*_ (% *I*_*ref*_)	0.01	0.01	0.03	0.12
Harmonics around 6*f*_*sw*_ (% *I*_*ref*_)	0.01	0.02	0.02	0.09
THD of *i*_*g*_ (%)	2.15	2.14	4.34	1.94

### 4.1. Simulation results

**Fig 9:**
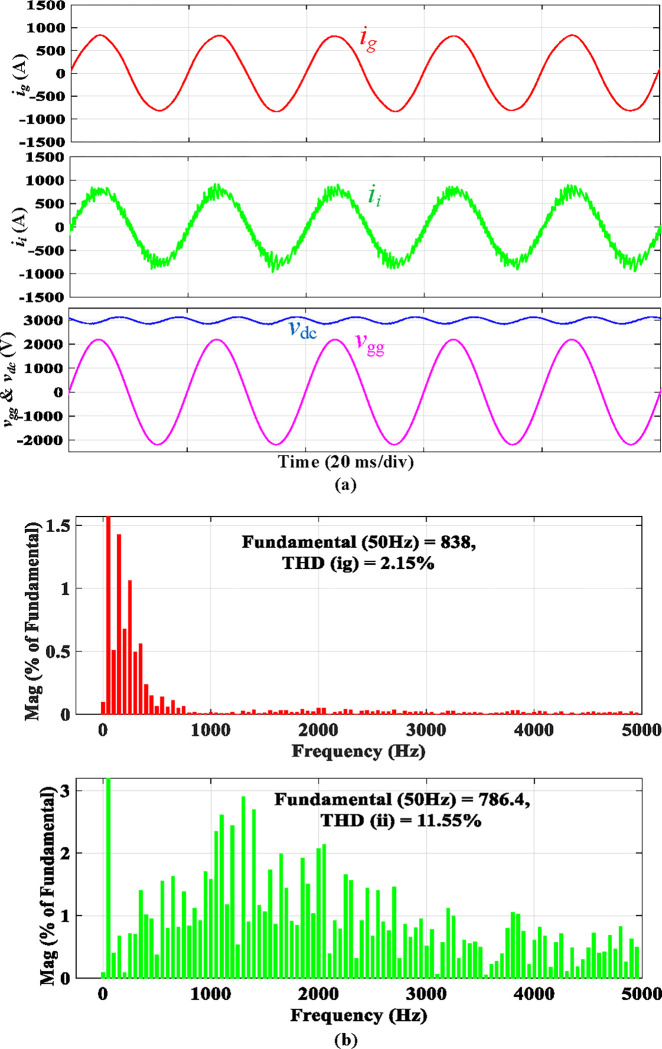
Simulation results using integrated *LLCL* filter.

The simulated steady-state waveforms of *i*_*g*_, *i*_*i*_, *v*_*gg*_, and *V*_*dc*_ with the proposed integrated *LLCL* filter are depicted in Fig 9(A). It is shown that *V*_*dc*_ here approximates 3000 V, and the error is lower than 150 V (5%) due to the controller the voltage loop. In the steady state, *i*_*g*_ is adequately filtered to be extremely sinusoidal. Fig 9(B) shows that its THD is just 2.15%, which is very low. This is mainly due to the proposed *LLCL* filter’s capability to reduce the low-order harmonics and double switching-frequency harmonics to a combined attenuation of 0.02%. With fourfold and sixfold switching-frequency harmonics of 0.01% and 0.01% of the fundamental current, the harmonic currents on the grid-side inductor are likewise attenuated satisfactorily. In this situation, all harmonic currents can be limited to less than 0.3%, which satisfies Table 3 displays the switching harmonics composition.

**Fig 10:**
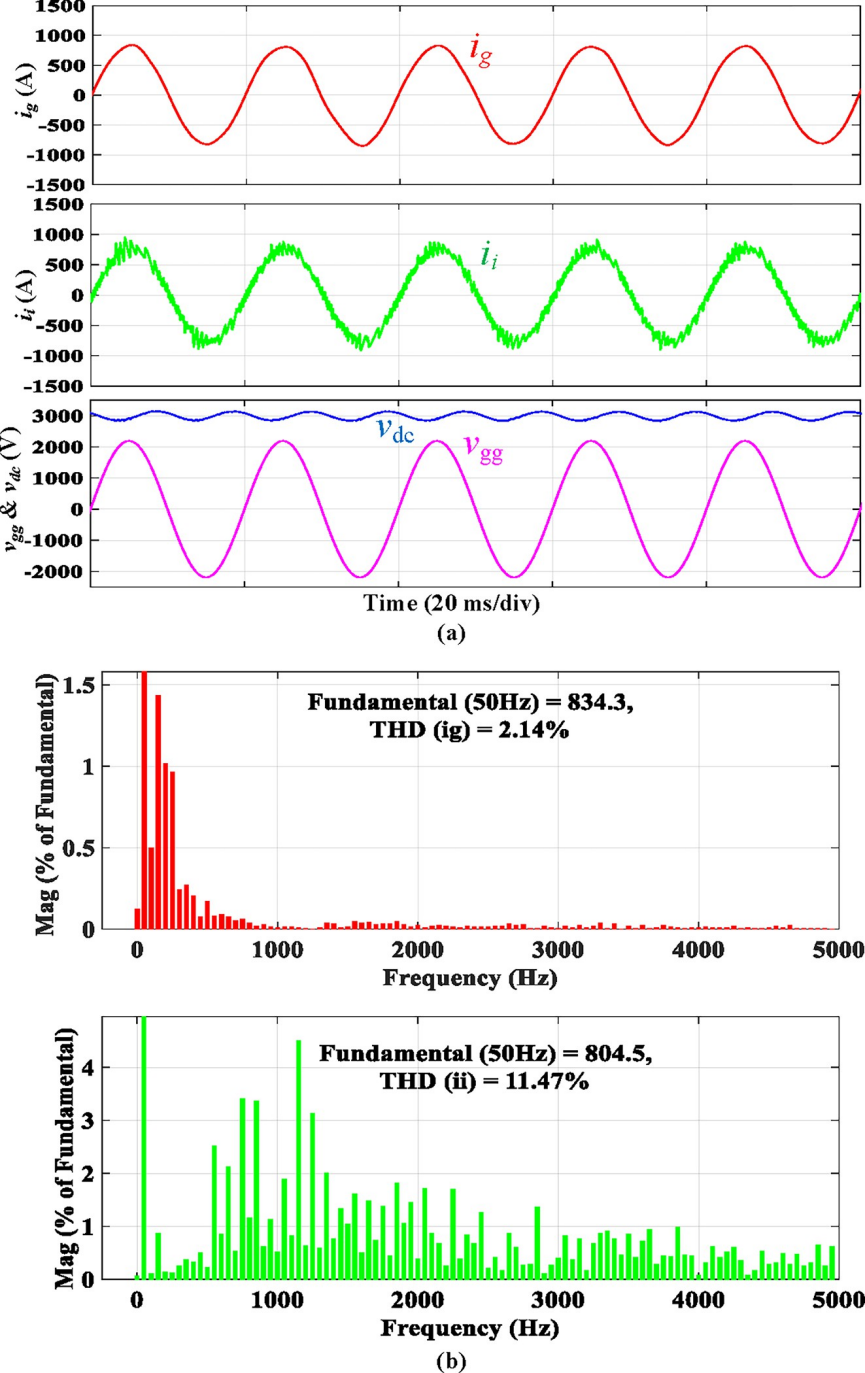
Simulation results using discrete *LLCL* filter.

For comparison, Fig 10(A) presents the simulated waveforms of a traction rectifier with a discrete *LLCL* filter. This section compares the proposed integrated filter’s performance to its discrete equivalent. As a result, Figs 9 and 10 indicate that the discrete *LLCL* filter likewise underwent the same test. When using the discrete *LLCL* filter in place of the integrated *LLCL* filter, the grid-side current waveform *i*_*g*_ still maintains a sinusoidal shape, proving that the compensation of harmonics at low frequencies is unaffected. Furthermore, the harmonics at 2*f*_*sw*_ of *i*_*g*_ are significantly decreased, as seen in Fig 10(B). In addition, the *i*_*g*_ harmonics over the double switching frequency are effectively suppressed. The fourfold sixfold switching-frequency harmonics are 0.01% and 0.01%, respectively, of the fundamental component, much lower than the limits. Table 3 shows that the specified *LLCL* filter can meet IEEE criteria with a grid-side current THD of 2.14% but with more extensive filter components. As demonstrated, the performance of the proposed filter, in general, is comparable to that of the discrete *LLCL* filter, indicating that it is effective with a small size.

**Fig 11:**
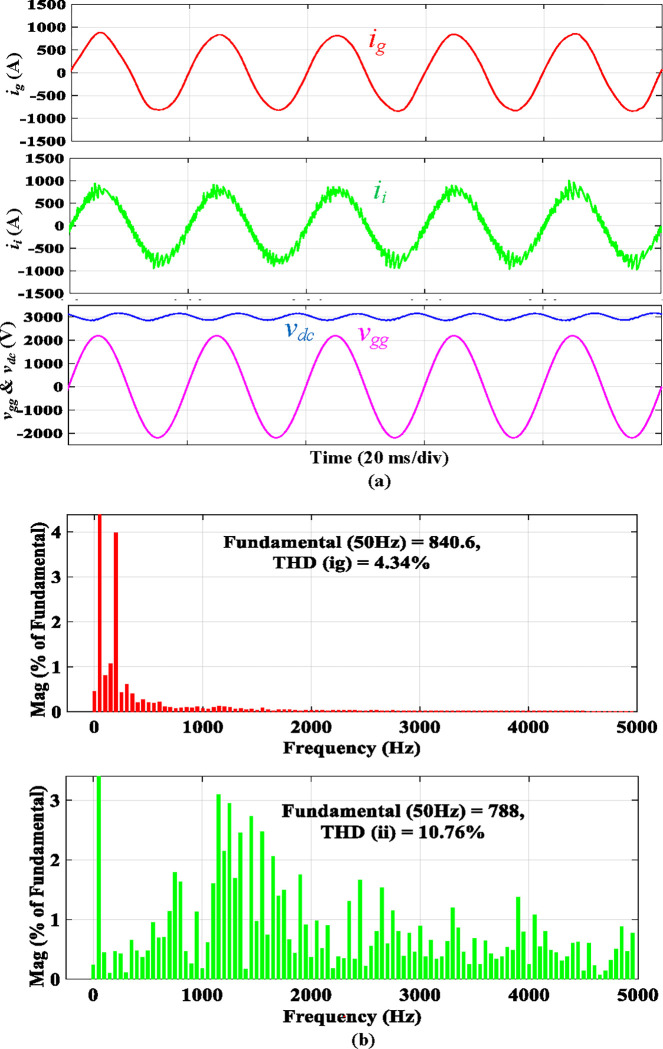
Simulation results using discrete *LCL* filter.

Fig 11 presents the simulated results of the traction PWM rectifier using a traditional *LCL* filter. The waveforms are presented in Fig 11(A), and the harmonic spectra of *i*_*g*_ and *i*_*i*_ are demonstrated in Fig 11(B). As seen in Fig 11(B), the 3^rd^ harmonic of *i*_*g*_ is approaching the threshold of 4.00% of the fundamental component. This unipolar modulated traction converter’s somewhat inferior performance can be attributed to its relatively low switching frequency and tiny filter inductances. THD of *i*_*g*_ is 4.34%, less than the allowable level. The proposed filter only needs one magnetic core, ‎while the discrete *LCL* one needs two cores.‎

**Fig 12:**
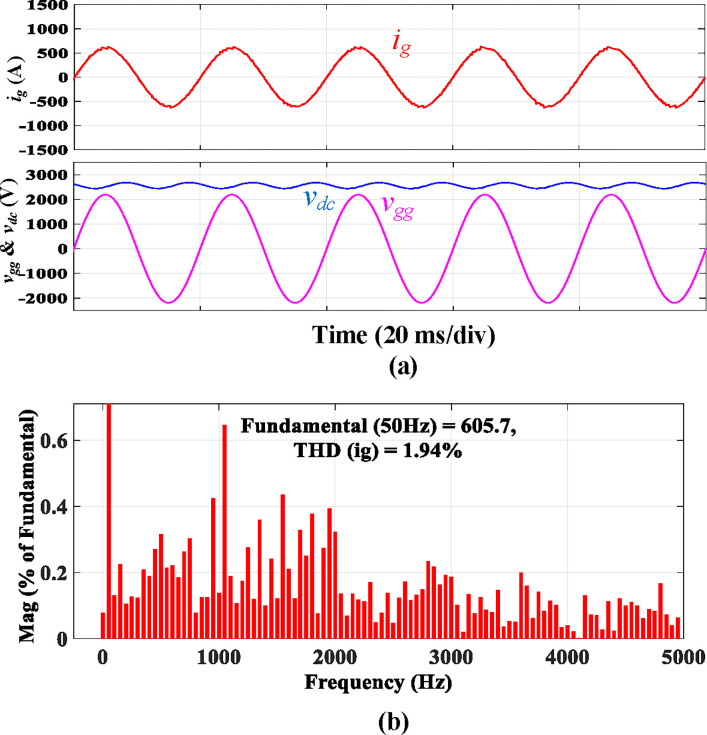
Simulation results using discrete *L* filter.

The simulated results of a traction rectifier with an *L* filter are depicted in Fig 12(A). Only the simulated results for *i*_*g*_ are presented because the simulations for *i*_*i*_ are identical. Although *i*_*g*_ is filtered to a sinusoidal shape and has the same phase of *v*_*gg*_, as shown in Fig 12(B), a current spiking at the 39^th^ harmonic, near 4*f*_*sw*_, is observed. The grid code will be broken as a result. Moreover, the *L* filter will cause the dc-link voltage to decrease to around 2560 V, which will harm the converter’s performance and increase the risk of traction blockage. The 2560 V dc-link voltage is not allowed in real-world operation. As a result, more research on this problem is recommended.

As seen in Table 3, although the THDs of the four filters are satisfied at less than 5%, the required inductance of the output filter differs. Furthermore, the first two filters’ switching current harmonics at the *LC*-trap frequency are equal, supporting the viability of the presented method. Moreover, in comparison with the discrete *LLCL* filter and the discrete *LCL* filter, two magnetic cores and one magnetic core, respectively, are saved. As a result, the proposed filter minimizes expenses and size.

When comparing thoroughly, it is essential to consider the inductors’ power losses, even if the integrated *LLCL* filter is less in size and weight than the discrete *LLCL* and *LCL* filters. Since the *L* filter is unstable, it would not be taken into consideration hereunder. The detailed analysis of the inductors’ power losses, which has previously been explored ‎in previous research [11], is not included here since this paper’s main emphasis is on the *LLCL* filter’s magnetic integration and usage in traction inverters. Inductors often lose power as a result of core and winding copper loss. Due to the numerous switching harmonics in the inverter output current *i*_*i*_ and voltage *v*_*in*_, the inductors’ power losses are not easily computed or measured.

To assess the inductors’ power losses in the integrated *LLCL*, discrete *LLCL*, and *LCL* filters, the system efficiency is estimated under similar circumstances. When working under a unit factor, *P*_*in*_ denotes the input active power calculated using *P*_*in*_ = *V*_*gg*_*I*_*g*rms_, while *P*_*o*_ denotes the output active power computed using *P*_*o*_ = *V*_*dc*_*I*_*dc*_. The ratio of *P*_*o*_/*P*_*in*_ is utilized to evaluate the efficiency of the system. The average values of *i*_dc_ and *v*_*dc*_, which may be determined using calculations or direct measurements, are *I*_dc_ and *V*_*dc*_. The RMS values of *i*_*g*_ and *v*_*gg*_ are *I*_*g*rms_ and *V*_*gg*_. To determine the power losses, *P*_*o*_ would be subtracted from *P*_*in*_. Although these losses seem high compared to the total power, they are much less than the allowed limits. The system efficiency of the integrated *LLCL* and discrete *LLCL* and *LCL* filters, if tested under conditions similar to full load, are 97.47%, 97.54%, and 97.37%, respectively. Since the efficiency is generally higher than 97.3%, there are not many power losses in the system. Thereby inferring that no additional countermeasures for power losses are not needed.

**Fig 13:**
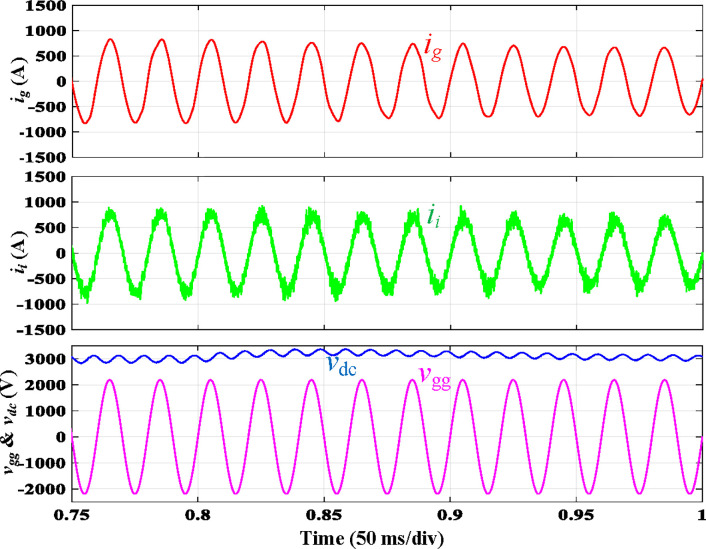
Simulation results of step-up load variation.

The proposed filter is subjected to load variation testing, and Fig 13 shows the associated simulated waveforms. The load was raised to 125% of its rated value (10 → 12.5) at t = 0.8 s. The entire fluctuation length was 0.13 s, which shows that the filter was designed well.

**Fig 14:**
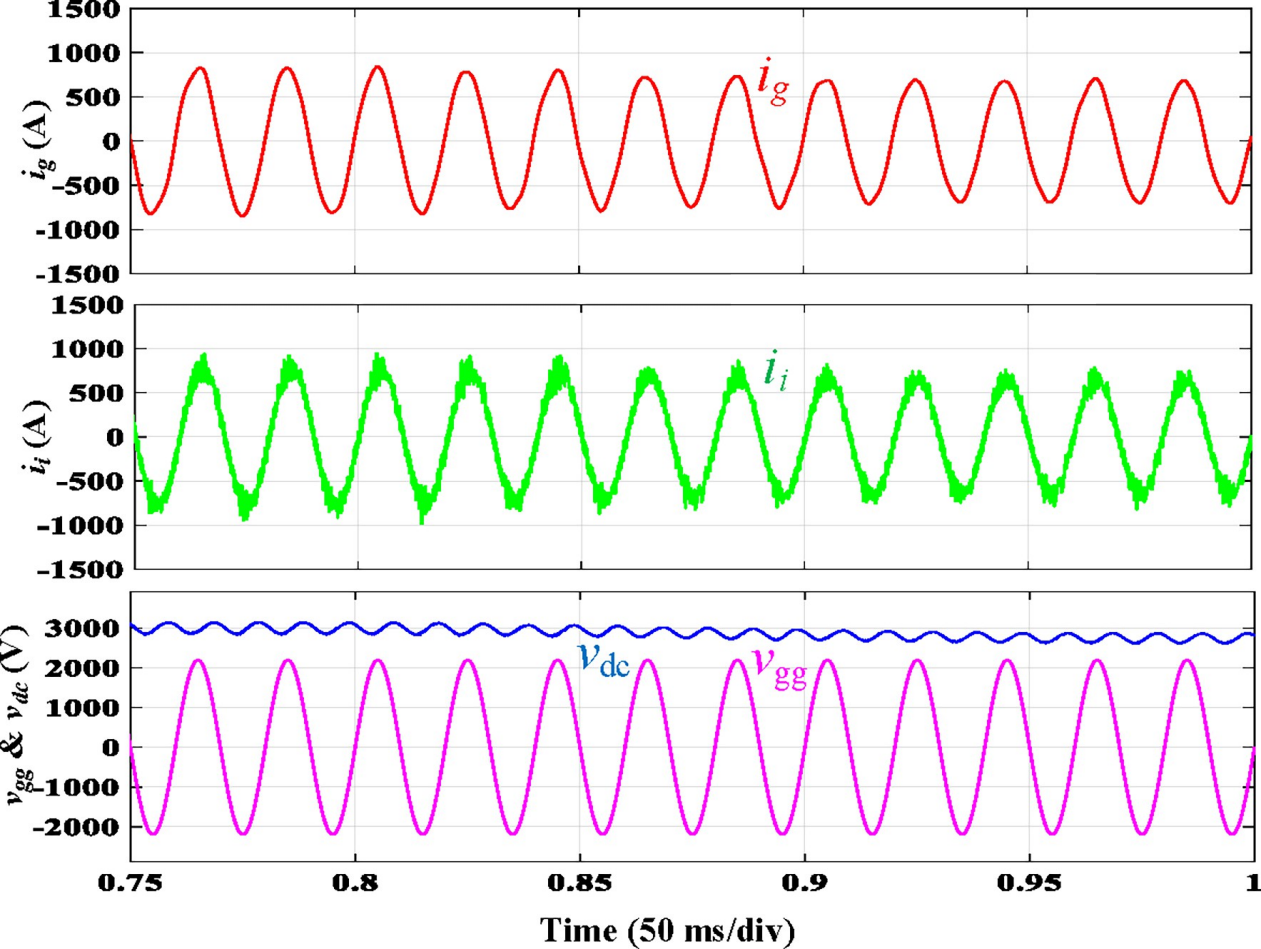
Simulation dynamic waveforms.

The simulation dynamic results of an integrated *LLCL* filter when the *V*_*dc*_ was suddenly stepped down by 300 V (3000 → 2700 V) at t = 0.8 s are shown in Fig 14. The presented method exhibits a well-designed filter in the dynamic phase and has strong harmonic switching elimination capabilities. Even though the filter experiences few transients, it finally stabilizes at its setpoint. The current requires about 0.12 s to follow its reference, even if it necessitates a longer time for the system to react dynamically.

The above results demonstrate good filtering performance, slight voltage variation, appropriate stability, quick dynamics features, and excellent resistance to the grid impedance of the proposed filter. The *LCL* filter also exhibits poor performance for harmonics at low frequencies. Furthermore, a significant size is required for the discrete *LLCL* filter. As a result, choosing the integrated *LLCL* filter is driven by its advantages over the alternatives.

Since the integrated *LLCL* filter possesses a sturdy structure, it may be inferred that it is appropriate for traction networks. The above simulation results are consistent with the above parts’ theoretical analyses.

### 4.2. HIL experimental results

**Fig 15:**
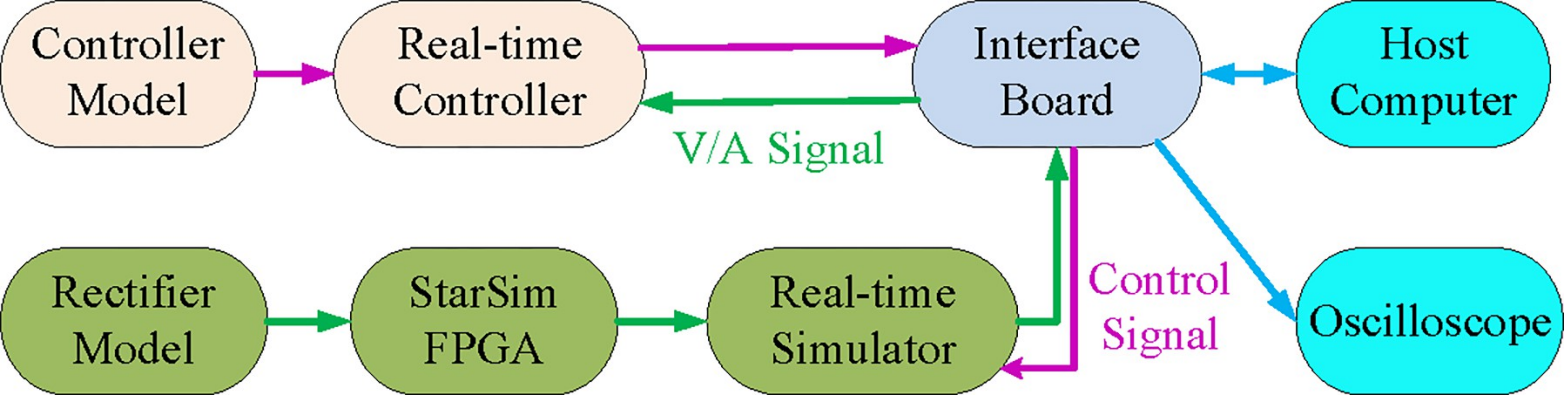
HIL experimental platforms schematic diagram.

The experiments are also carried out on the HIL platform for further verification. The HIL experimental platform has been set for verifying the superiority, feasibility, and applicability of the presented filter. The main advantage of the HIL experimental platform is that the actual model may be assessed without physical hardware, as shown in Fig 15 [75]. Another advantage is that the designer need not rely on environmental or naturalistic tests. The HIL platform’s use is practical and economical because the models can simulate plants. Using HIL, it would be feasible to reduce the expense of hardware verifications and the time and effort required for developing modifications in various applications [76,77].

In addition, difficulties in recognizing and redesigning are easy, thanks to HIL experiments. Furthermore, it enables real-time tests to progress through the entire process faster than hardware tests. HIL is also more accurate and less expensive than physical testing because of its ability to be developed and executed on a schedule.

This method has shown considerable potential for both industry and academics because of HIL’s ability to provide riskless equipment and a speedy prototype procedure in research of engineering [4,8,11,31,35,60,75–84]. If a system with high power, such as TPSS, would be included, HIL tests may offer a safe testing environment. HIL is an attractive technique that offers the capabilities to test design methodology in a scenario of extensive systems with many complicated independent models that have high switching frequencies or quick dynamic behavior [4,8,31,60,76,82,84]. In addition, HIL is a contemporary technology frequently utilized for power electronic system design and validation. To address the problems of difficulty, complexity, and expense, HIL has been used to test power converters [75,77–81]. Results have demonstrated that HIL verification is a useful method for assessing the grid-connected passive-filtered inverter [8,11,31,35,78,79,83].

It should be made clear that the presented filter is currently in the research phase. It will be more cost effective to design and test the proposed filter utilizing the HIL platform in this step rather than the physical filter design that could happen in the subsequent phases because it is impossible to build an actual TPSS at laboratory tests. It is important to note that the HIL technology is an essential verification tool for new design techniques in large systems like TPSS. This technique allows for the examination of the accuracy and efficacy of the system under study with no spending expense of actual systems [4,8,31,60,76,82,84]. The standard method in the HIL technique is used in this work to model the proposed filter. Utilizing this standard method for the examined circuit, it is believed that the HIL technique produces relevant results that are extremely near to those of the actual tests, as in the systems presented in [8,11,31,35,78,79,83].

**Fig 16:**
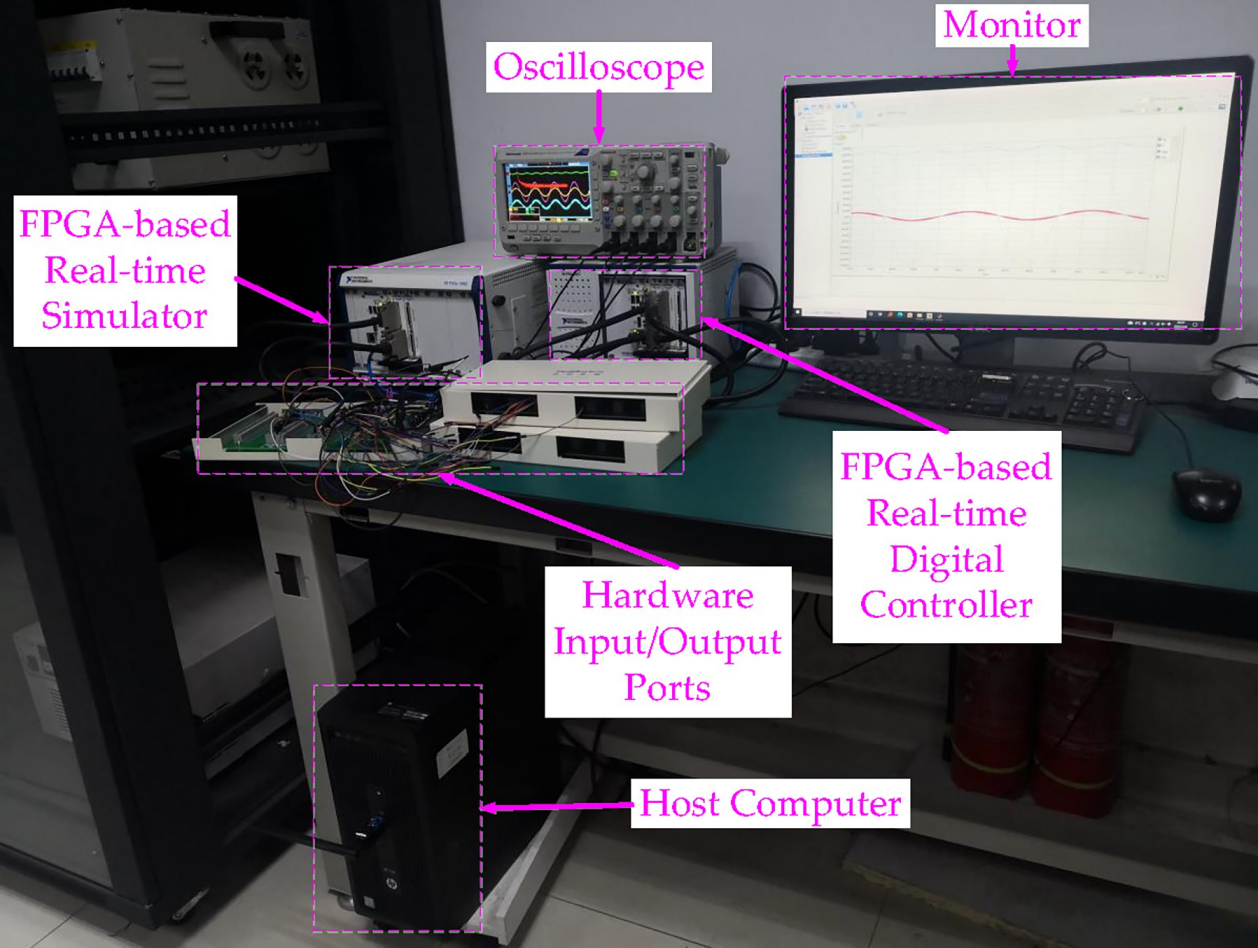
Configurations of HIL experiments.

A fast control prototyping unit developed by StarSim modeling and integrated into the NI PXle-8821-FPGA-7868R real-time controller (RTC) and the NI PXle-8821- 7846R real-time simulator (RTS) are all part of the HIL [84], as shown in Fig 16. The HIL also includes a power system emulating unit, hardware input/output ports, an oscilloscope, and a host computer. The translucent backboard of the NI PXle-1082 has eight slots and offers exceptional performance and output power.

Moreover, it provides a high degree of durability, which reduces the time needed for repairs [77]. It has improved synchronization capabilities and matches the OXI-5 PXI hardware requirements. The NI PXle-1071, on the other hand, is a 4-position PXI platform backboard, robust, and rack-attachable, indicating that its basic design was developed for optimum use and adaptability ‎[84].

MATLAB/Simulink can be utilized for programming the control system, where the fixed-step solver can be used. Using the modeling program StarSim, the grid-connected rectifier with filter and control system models are uploaded into the HIL. Executing the grid-connected rectifier with filter model and control system model in RTS and RTC, respectively, results in constructing a closed loop. Like the simulation verification, identical parameters are used. The oscilloscope and monitor could be used for tracking the voltage and current waveforms. The oscilloscope may also provide data, such as S1 Data, for the experimental current/voltage waveforms, which might then be uploaded to the MATLAB/Simulink program and examined with the Powergui FFT Analysis Tool.

**Fig 17:**
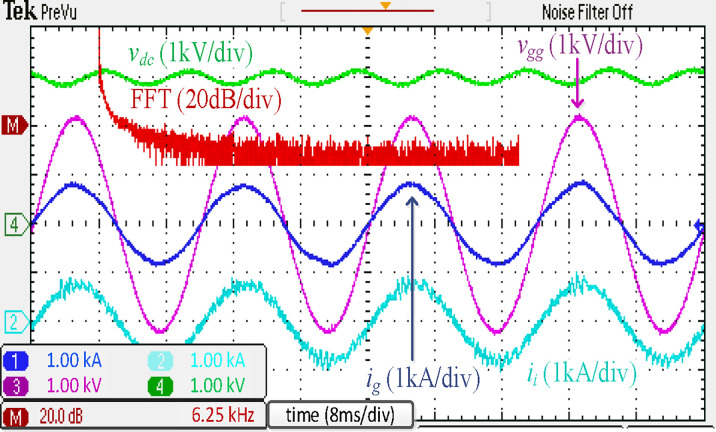
Experimental results using integrated *LLCL* filter.

Fig 17 depicts the experimental results of a traction inverter with an integrated *LLCL* filter. These results match the simulation results in Fig 9. The prominent switching harmonics were mostly eliminated because the *LC*-trap frequency is adjusted at 2*f*_*sw*_. To meet the requirements of grid regulations, as tabulated in [70], it is possible to significantly reduce the THD of *i*_*g*_.

**Fig 18:**
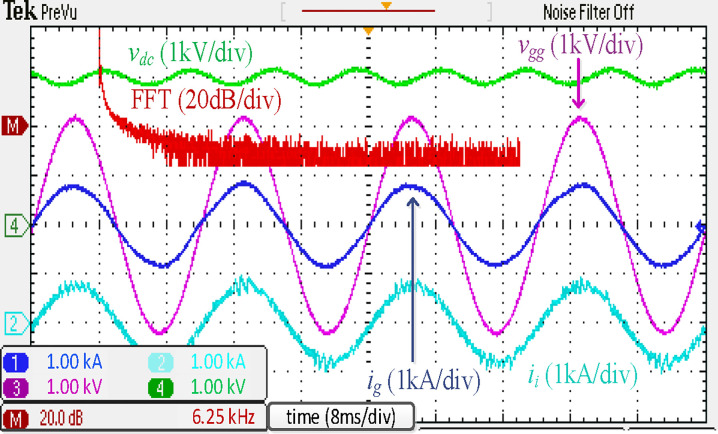
Experimental results using discrete *LLCL* filter.

The experimental results of the discrete *LLCL* filter are shown in Fig 18. These results demonstrate that harmonics of *i*_*g*_ at the prominent switching frequency would not surpass the acceptable threshold. This filter’s *LC*-trap is the fundamental reason for this good performance, supporting the theoretical analysis and simulation findings in Fig 10.

**Fig 19:**
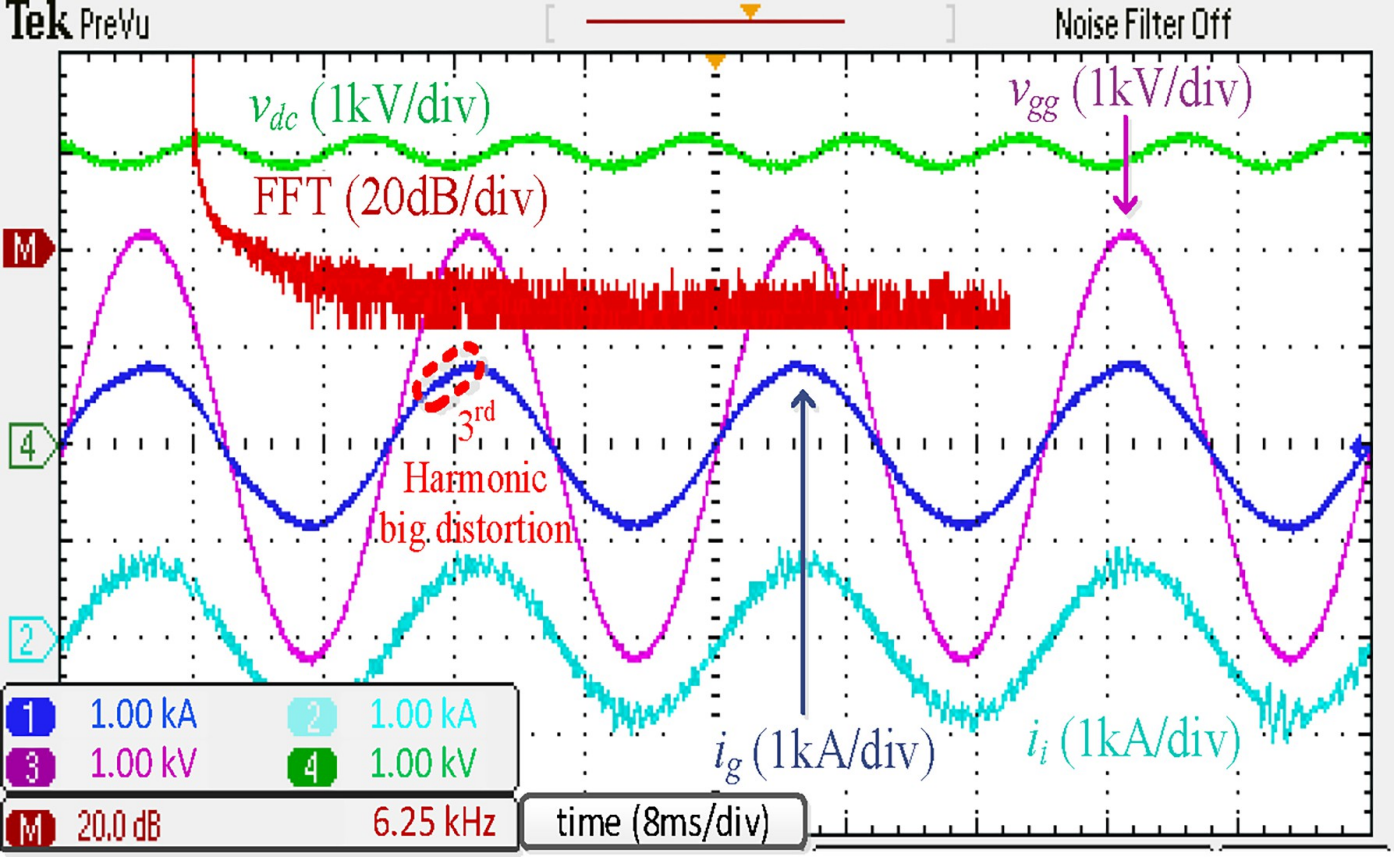
Experimental results using discrete *LCL* filter.

Fig 19 illustrates the experimental results of the discrete *LCL* filter. The low-frequency harmonics of *i*_*g*_ at the were found to almost surpass the requirements. This problem is caused by the unipolar modulated traction rectifier’s low switching frequency and tiny filter inductors. As in the simulation results in Fig 11, the THD of *i*_*g*_ could be seen as less than the acceptable levels.

**Fig 20:**
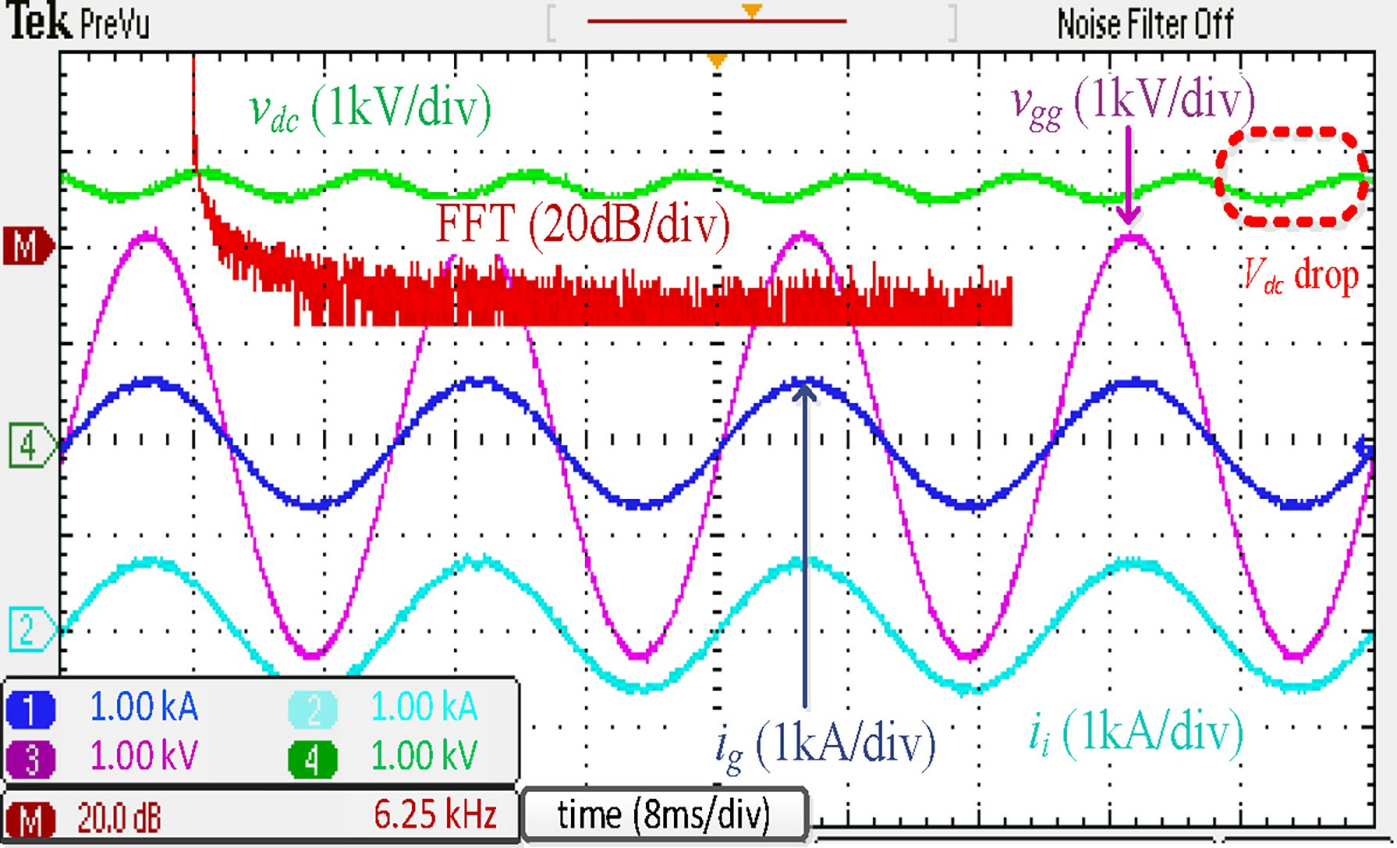
Experimental results using traditional *L* filter.

Fig 20 displays the experimental results using the conventional *L* filter. Despite the *i*_*g*_ being filtered to a sinusoidal shape and in phase with *v*_*gg*_, a current spiking in the 39^th^ harmonic, beside 4*f*_*sw*_, was seen. There will be a breach of the grid codes as a result of this. In addition, *V*_*dc*_ would decrease to around 2560 V, like the simulated results presented in Fig 12. Thus, this situation may lead to poor converter performance or traction blockage. In real life, a *V*_*dc*_ of 2560 V would not be allowed. Therefore, additional research needs to investigate the problem more thoroughly.

**Fig 21:**
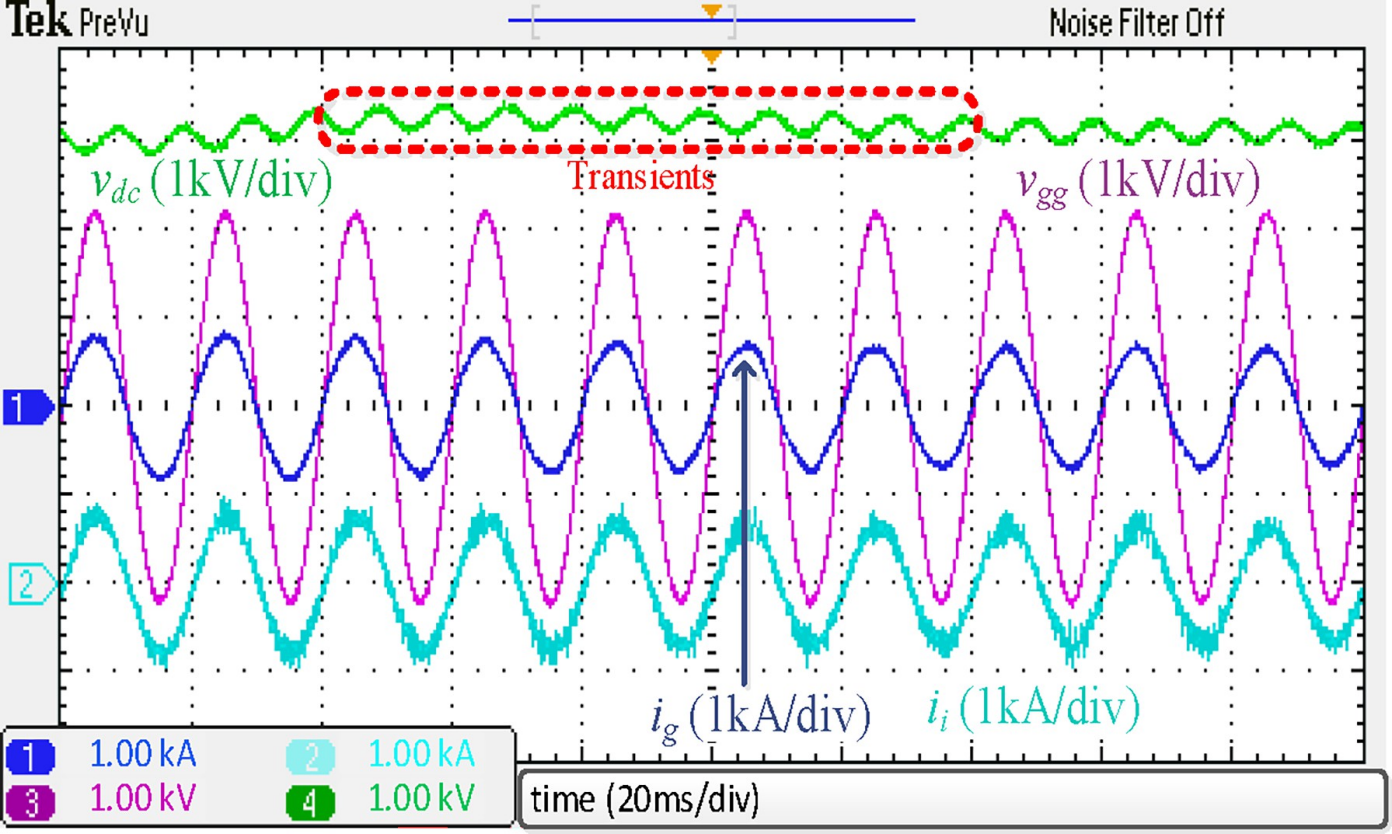
Experimental results of step-up load variation.

The experimental results of an integrated *LLCL* filter with a 25% *R*_*dc*_ increment in varying load conditions are shown in Fig 21. The system can function appropriately in the face of transient occurrences.

**Fig 22:**
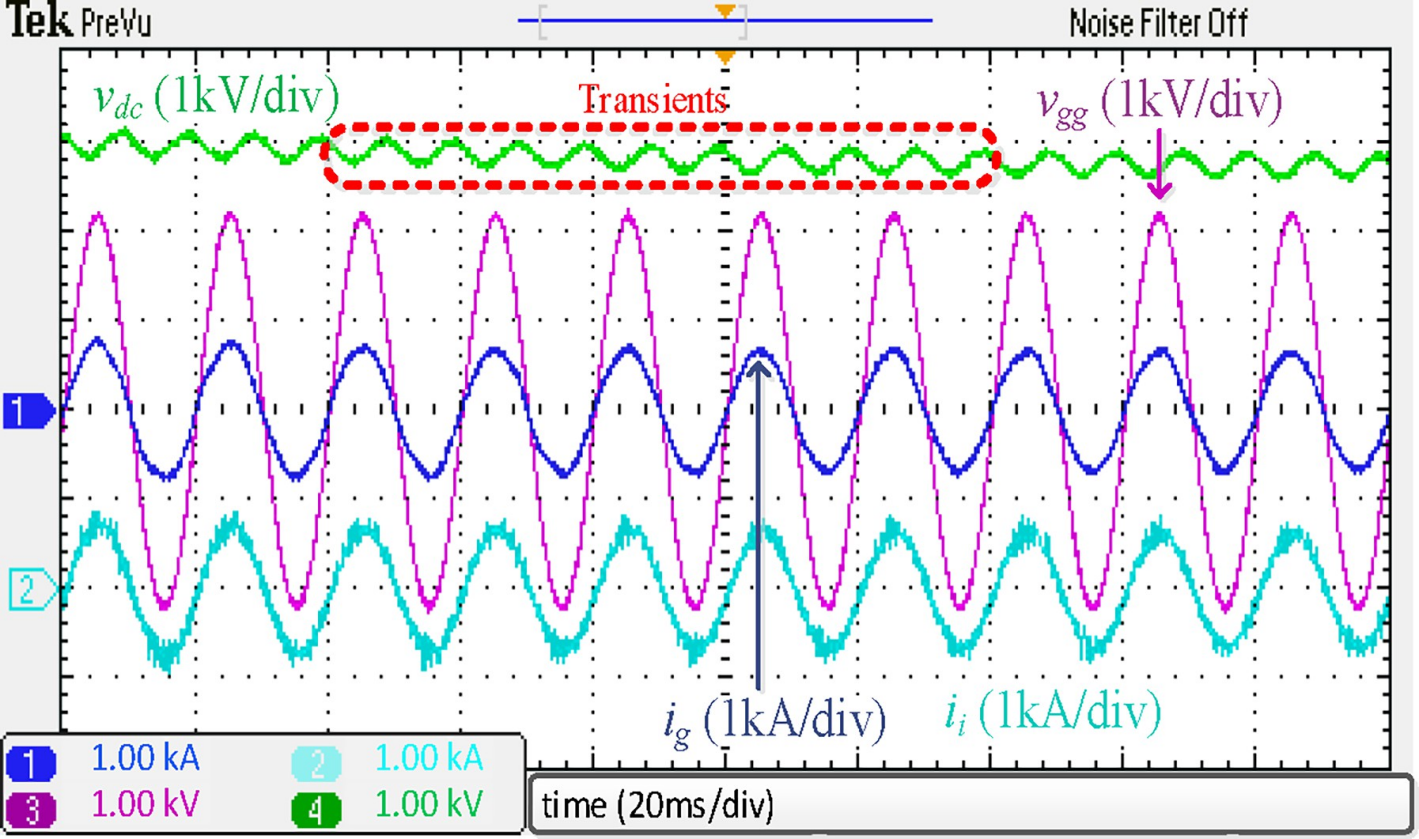
Experimental dynamic results.

The dynamic test was performed for the integrated *LLCL* filter, where Fig 22 shows the experimental results of a 300 V step-down variation in the *V*_*dc*_ (3000 → 2700 V). As can be observed, the waveforms are comparable to those simulated in Fig 14, proving that the filter can provide both stability and switching harmonic attenuation. The integrated *LLCL* filter is seen to have certain transient moments and then return to its setpoint with no fluctuation, which verifies the system’s robustness. Even though the transient phase only lasted for around 0.12 s, the system’s dynamic responsiveness needed a brief time till the current began following its reference.

The experimental results are consistent with the simulated and theoretical analyses discussed in the previous sections. These simulation and experimental results verify the accuracy of the theoretical analysis and show that the proposed integrated *LLCL* filter maintains the benefits of the discrete *LLCL* and *LCL* ones while minimizing their drawbacks. The results demonstrate that the presented parameter design approach is successful, the proposed parameter robustness analysis technique is accurate, and the presented filter performs comparably to the discrete *LLCL* filter. The integrated *LLCL* filter is also flexible, improve filtering performance in the steady state, and effective in load variations and dynamics working conditions.

## 5. Conclusion

To reduce the size and weight of the inductors because high-speed trains have minimal space, an integrated *LLCL* filter with resonance frequency beyond the Nyquist frequency is proposed in this paper for traction rectifiers to suppress the dominant current switching harmonics. Based on the traditional *LCL* filter, an *LC*-trap could be created by introducing a coupling inductance into the filter capacitor branch via the magnetic coupling of the inverter-side and grid-side windings. It is possible to tune this *LC* trap to a specific harmonic frequency using a stepwise design method. The proposed filter can save two magnetic cores and achieve an identical harmonic suppression performance as the discrete *LLCL* filter. Furthermore, the proposed filter is with a magnetic core structure similar to the integrated *LCL* filter but performs better in harmonic suppression. The presented filter has been provided with a detailed step-by-step design method to facilitate the parameter choices. The developed filter could also withstand the grid impedance changes. After completing MATLAB/Simulink simulations and HIL experimental models, the verification results were provided to verify the following merits of the integrated *LLCL* filter:

1. The proposed filter has fewer discrete passive elements compared to the discrete *LLCL* and *LCL* filters, which enables decreasing the cores number of 1 and 2 respectively.
2. The proposed approach provides good harmonics reduction and resonance clearing compared to conventional passive filters, leading to a grid-side current THD value ‎of 2.15%.
3. The method made it possible for the flexibility of filter design and efficacy of magnetic integration, which can provide a reference for designing new passive filters.
4. It further achieved durability and stability in transient occurrences, with an entire fluctuation length of only around 0.12 s.

However, the passive-filtered traction converters must maintain grid connectivity and carry out fault ride-through in the event of grid faults; else, system instability would occur. Hence, this instability problem and its solution must be adequately researched in the future.

## Supporting information

S1 Data Processing results waveform data using StarSim. (CSV)

S1 Appendix A list of the coefficients in (11). (DOCX)
