# Supplementary material for: High-frequency harmonics suppression in high-speed railway through magnetic integrated LLCL filter
Source: PLoS One. 2024 Jun 3;19(6):e0304464. doi: 10.1371/journal.pone.0304464 (PMC11146739; doi:10.1371/journal.pone.0304464)
Supplement: S1 Appendix — (DOCX) [file pone.0304464.s002.docx]

Appendix

The following is a list of the coefficients in (11):

$$a_{4}=C_{f}C_{g}M_{ig}(L_{g}-M_{ig})$$

$$a_{2}=C_{f}M_{ig}+C_{g}(L_{g}-M_{ig})$$

$$b_{5}=C_{f}C_{g}(L_{g}-M_{ig})(L_{i}M_{ig}-M_{ig}^{2}+L_{i}L_{s})$$

$$b_{3}=C_{f}(L_{i}-M_{ig})(L_{g}-M_{ig}+L_{s})+C_{g}(L_{g}-M_{ig})(L_{i}-M_{ig}+L_{s})+C_{f}M_{ig}(L_{i}+L_{g}-2M_{ig}+L_{s})$$

$$b_{1}=L_{i}+L_{g}-2M_{ig}+L_{s}.$$
